# Human resource challenges in health systems: evidence from 10 African countries

**DOI:** 10.1093/heapol/czae034

**Published:** 2024-05-06

**Authors:** Ashley Sheffel, Kathryn G Andrews, Ruben Conner, Laura Di Giorgio, David K Evans, Roberta Gatti, Magnus Lindelow, Jigyasa Sharma, Jakob Svensson, Waly Wane, Anna Welander Tärneberg

**Affiliations:** Department of International Health, Johns Hopkins Bloomberg School of Public Health, 615 N. Wolfe St., Baltimore, MD 21205, United States; World Bank, 1818 H Street, NW Washington, DC 20433, United States; World Bank, 1818 H Street, NW Washington, DC 20433, United States; World Bank, 1818 H Street, NW Washington, DC 20433, United States; Inter-American Development Bank, 1300 New York Avenue NW, Washington, DC 20577, United States; World Bank, 1818 H Street, NW Washington, DC 20433, United States; World Bank, 1818 H Street, NW Washington, DC 20433, United States; World Bank, 1818 H Street, NW Washington, DC 20433, United States; Stockholm University, Institute for International Economic Studies, Universitetsvägen 10A, Stockholm 114 18, Sweden; World Bank, Rue Washington, Abidjan, Côte d’Ivoire; Centre for Economic Demography and Department of Economic History, Lund University School of Economics and Management, Scheelevägen 15B, Lund 223 63, Sweden

**Keywords:** Human resources, quality of care, developing countries, health systems research, international health, knowledge, staffing levels

## Abstract

Sub-Saharan Africa has fewer medical workers per capita than any region of the world, and that shortage has been highlighted consistently as a critical constraint to improving health outcomes in the region. This paper draws on newly available, systematic, comparable data from 10 countries in the region to explore the dimensions of this shortage. We find wide variation in human resources performance metrics, both within and across countries. Many facilities are barely staffed, and effective staffing levels fall further when adjusted for health worker absences. However, caseloads—while also varying widely within and across countries—are also low in many settings, suggesting that even within countries, deployment rather than shortages, together with barriers to demand, may be the principal challenges. Beyond raw numbers, we observe significant proportions of health workers with very low levels of clinical knowledge on standard maternal and child health conditions. This study highlights that countries may need to invest broadly in health workforce deployment, improvements in capacity and performance of the health workforce, and on addressing demand constraints, rather than focusing narrowly on increases in staffing numbers.

Key messages

- This study analysed health worker surveys from 10 countries in sub-Saharan Africa for a deeper understanding of human resource challenges.
- Average staffing across facilities is far below the stated staffing norms for each country.
- Half of health centres and health posts have one or fewer clinical staff assigned to them.
- Staffing is even lower when adjusted for health worker absence from facilities, which is highest in small facilities and public facilities.
- Massive within-country variation in caseload suggests that staffing problems may be solved in part by reallocation of clinical staff.
- Health workers lack basic clinical competencies, caseloads are imbalanced, and there is substantial absence of workers from health facilities.

## Introduction

For decades, experts both on the African continent and those in the international community have highlighted a shortage in human resources for health in Africa (Huddart and Picazo, 2003; Gomes Sambo, 2005; World Health Organization, 2006; Elkhalifa, 2014; Fieno *et al*., 2016). One analysis projected a shortfall of six million health workers across the continent by 2030 (Tulenko, 2016). African countries, on average, have fewer medical workers per capita than any other region in the world.^1^ Yet while a shortage of health workers is undoubtedly a concern, it is not the only human resource challenge to Africa’s health systems. Recent analysis has highlighted others, including low quality of health worker knowledge (Daniels et al., forthcoming; Di Giorgio *et al*., 2020; Okeke, 2021) and low access to and utilization of health services (Abekah-Nkrumah, 2019). Complicating the debate is the fact that all of these factors are endogenous: understaffed or under-skilled health facilities may result in low service utilization, and low service utilization may affect the number of staff assigned to a facility.

This paper draws on newly available data from 10 countries in sub-Saharan Africa (SSA) to answer four key questions. First, how many health workers are there? Second, are health workers present and what are the implications of worker attendance on effective staffing levels? Third, how heavy are health workers’ caseloads? Fourth, how competent are health workers? We use the Service Delivery Indicators (SDI) surveys—facility surveys that are designed to be nationally representative and to provide comparable data across several countries—to demonstrate how challenges tend to be either consistent or divergent across settings. In this study, we focus on within-country description and cross-country comparison to answer these staffing questions and consider the implications for effective healthcare delivery in low- and middle-income settings across Africa.

We add to a rich, existing literature on the crisis in human resources for health (George *et al*., 2017). Relevant previous analyses have focused on identifying cross-country gaps in health workforce either throughout the world (World Health Organization, 2006) or across Africa (e.g. Lawal, 2019; Ahmat *et al*., 2022). Others have focused on the health workforce challenges unique to individual country contexts. For example, Okeke (2021) experimentally tests whether staffing shortages or staff ability is the binding constraint in Nigeria, finding evidence in favour of ability, and Fieno *et al*. (2016) examine the political economy of staffing challenges in Ethiopia, suggesting that political will, strong state capacity, and additional resources can enable increasing the supply of health workers.

Finally, a key strand of the literature discusses potential contributing factors to the human resources for health challenges and possible interventions to address them, with significant attention given to the issue of trained health workers leaving the country in search of better work conditions (Liese and Dussault, 2004; Lawal, 2019; Ivins *et al*., 2022). Kinfu (2009) explores the range of reasons that health workers across Africa attrit from the profession, including retirement, mortality and out-migration. George *et al*. (2012) show how, in South Africa, merely examining the numbers masks other staffing challenges, such as training health workers in the wrong specialities. However, few previous analyses have had the data to look more comprehensively at staffing shortages in Africa by providing comparable analysis across countries that highlights both high-level country averages and within-country variation.

## Methods

### Country selection

The SDI survey has been implemented across numerous countries in SSA over the last 10 years. This study draws on data from the SSA region: Kenya (2018), Madagascar (2016), Malawi (2019), Mozambique (2014), Niger (2015), Nigeria (2013), Sierra Leone (2018), Tanzania (2016), Togo (2013) and Uganda (2013). We included all SDIs conducted in SSA to shed light on the state of quality of care in this region. One survey (Senegal 2010) was excluded as it was a pilot survey and not directly comparable, and the most recently completed SDIs were not included as the data were not yet available. For countries that have implemented multiple rounds of the SDI, we used the most recent survey (i.e. Kenya 2018 and Tanzania 2016). SSA is a diverse region composed of low, lower-middle, upper-middle and high-income countries. The selected countries are a diverse group by size, population, income level and key health indicators (Supplementary Table S1). On average, these 10 countries have a similar average income to the region as a whole. On average, health outcomes tend to be worse in these 10 countries than in the region as a whole, with lower life expectancy, higher infant and maternal mortality and higher malaria risk.^2^

### Data: the SDI

The SDI health surveys, co-led by the World Bank and various country governments, are health facility assessments used in low- and middle-income countries to generate nationally representative data on health service delivery with a focus on primary healthcare service quality (Bold *et al*., 2010; 2011). Using standardized instruments adapted to each country context, the SDI collects both facility and health worker–level information that includes service availability and readiness, health worker capacity to diagnose and treat common illnesses and health worker presence through unannounced visits. Data are made publicly available by the World Bank through the SDI data repository (The World Bank, 2021).

The SDI country reports for the selected countries contain comprehensive information on each survey’s methodology and questionnaires. Briefly described, all surveyed facilities completed a facility inventory questionnaire. In addition, a health worker roster collected information on the total number of health workers at the facility and was used to randomly sample up to 10 health workers for follow-up on a second unannounced visit to the facility to assess health worker absences from the facility. Clinical vignettes were presented to up to 10 randomly selected health workers who conducted outpatient consultations at the facility.

The total study sample includes interviews from 79 441 health workers,^3^ observations of when health workers are not present at the facility from 35 896 health workers, assessments of competency from 15 207 health workers and audits from 8916 health facilities from across 10 countries in Africa. Details of the sample are listed in Table 1 with further disaggregation by country, facility type, managing authority, urban/rural and health worker cadre in Supplementary Table S2.

**Table 1.** Total number of facilities, health workers, health workers assessed for absence from facilities and health workers assessed for competency, by country

Country	Year	Total number of facilities	Total number of health workers—staffing	Total number of health workers—absenteeism	Total number of health workers—competency
Kenya	2018	3038	24 404	12 266	4485
Madagascar	2016	444	2200	1565	619
Malawi	2019	1106	13 290	4305	1522
Mozambique	2014	195	2972	936	694
Niger	2015	255	1331	661	514
Nigeria	2013	2385	21 318	9764	5014
Sierra Leone	2018	536	5055	2087	829
Tanzania	2016	383	5160	2119	498
Togo	2013	180	1364	917	303
Uganda	2013	394	2347	1276	729
Total		8916	79 441	35 896	15 207

### Empirical strategy

We estimated three dimensions of health workforce performance—availability, productivity and competency—each necessary to the achievement of improved service delivery and health outcomes (World Health Organization, 2006). To assess the availability of health workers, we examined staffing levels and health worker absences from facilities. Specifically, to assess staffing, a complete listing of staff working at the health facility, including each individual’s cadre, was collected through a staff roster questionnaire. Health worker absence from the facility was defined as the proportion of randomly selected health workers who were absent from the health facility during an unannounced facility visit who were scheduled to be present at the facility (this includes authorized and unauthorized absences). We calculated total clinical staff adjusted for health worker absence from the facility by taking the total number of clinical staff from the roster and reducing that number by the proportion of clinical staff absent, based on the results of the health worker absence assessment.^4^

To assess health worker productivity, we defined caseload as the number of outpatient visits recorded in outpatient records in the 3 months prior to the survey, divided by the number of days the facility was open during the 3-month period and the number of health professionals who conduct outpatient consultations. In some analyses, this indicator was adjusted for the average health worker absence at the facility level.^5^

To assess health worker competency, we examined diagnostic and treatment accuracy using data collected through clinical vignettes which are standardized clinical case simulations developed to assess the health workers’ ability to diagnose and treat common outpatient conditions (Das *et al*., 2008). Diagnostic accuracy was calculated as the percentage of vignettes for which the health worker gave the correct diagnosis while treatment accuracy was calculated as the percentage of vignettes for which the health worker gave the correct treatment. A combined measure of diagnostic and treatment accuracy was calculated as the percentage of vignettes for which the health worker gave the correct diagnosis and the correct treatment. Supplementary Table S3 provides a detailed description of the vignette requirements by condition used to calculate correct diagnosis and correct treatment.

Several additional facility-level and health worker–level measures were included in the exploration of the extent to which accounting for health worker characteristics explains differences in health worker performance. Infrastructure availability was calculated as the availability of three components: improved water source, improved sanitation and electricity. Equipment availability was calculated based on four items being available and functional: a thermometer, a stethoscope, a sphygmomanometer and a weighing scale. Detailed definitions for each measure can be found in Supplementary Table S4. Mean health worker absence from the facility and mean provider competency were calculated at the facility level for use in the facility-level regression model. Facility type was reviewed across countries and reclassified into three categories (hospital,^6^ health centre,^7^ and health post),^8^ while managing authority was reclassified into two categories (public and private/non-governmental organization (NGO)). Similarly, health worker cadre was recategorized into three categories (doctors/clinical officers, nurses/midwives and other)^9^ and health worker education into three broad categories (primary, secondary and post-secondary education).

Descriptive results, summarized as proportions and means, were calculated for all main measures and disaggregated by facility type, managing authority and urbanicity where applicable. For health worker–level analyses, results were also disaggregated by health worker cadre. For all analyses, survey weights were incorporated to account for the complex survey design.^10^ Descriptive analyses are presented separately for each country. In addition, we present the cross-country average calculated as the average of the 10 country estimates. We investigated the association of our key health workforce measures (health worker absence from the facility, caseload and diagnostic and treatment accuracy) with facility and health worker characteristics using multi-variable linear and logistic regression models with standard errors adjusted for clustering of health workers within facilities. The regression analysis was conducted on a pooled dataset of all countries except Uganda, which was omitted due to missing data on key regression variables.^11^ All analyses were conducted with Stata 15 and R-4.0.3 (Statacorp, 2017; R Core Team, 2022).

### Ethical approval

Each of the SDI surveys was carried out in collaboration with the Ministry of Health in each country. All survey participants consented to participate in the survey. Details regarding the ethics approval are available in each country survey report. This is a secondary analysis of SDI data.

## Results

### How many health workers are there?

To understand how many health workers (i.e. all cadres of staff in a facility) are deployed to facilities, we first looked at the distribution of health worker cadres by facility type (Table 2). We then looked at staffing patterns to understand the average number of clinical staff (i.e. cadres of health workers expected to provide clinical care including doctors, clinical officers, nurses and midwives) deployed per facility as well as the proportion of facilities with limited clinical staff (Table 3). We compared those levels with staffing norms for the countries (Supplementary Tables S5 and S6).

**Table 2.** Distribution of health worker cadres by facility type, by country (%)

	Kenya	Madagascar	Malawi	Mozambique	Niger	Nigeria	Sierra Leone	Tanzania	Togo	Uganda	All
Hospital											
Doctor/clinical officer	19.9	31.1	21.7	39.9	6.2	10.1	3.7	24.3	14.4	22.6	19.4
Nurse/midwife	45.4	46.6	57.7	37.7	65.1	31.9	58	43.3	45	44.3	47.5
Other	34.7	22.3	20.5	22.4	28.7	58	38.3	32.4	40.6	33	33.1
Health centre											
Doctor/clinical officer	18.9	32.5	5.1	26.8	2.9	1.3	0.2	20.5	13.4	18	14
Nurse/midwife	45.7	49	32.3	47.5	70.3	10.7	27.2	36.7	42.3	47	40.9
Other	35.4	18.5	62.5	25.7	26.8	88	72.6	42.8	44.3	35	45.2
Health post											
Doctor/clinical officer	17.1	3.1	2.1	NA	4.1	0.7	0	20.3	4	15.8	7.5
Nurse/midwife	47.1	76.7	17.7	NA	28.6	2.3	12.5	28.6	43.5	42.9	33.3
Other	35.7	20.2	80.2	NA	67.3	97	87.5	51.1	52.5	41.3	59.2

Note: Out of the 79 441 health workers in the dataset, 509 were missing information on cadre. These health workers come from 5 of the 10 countries in the analysis, and this represents <1% of the sample. We have therefore excluded these health workers from the analysis.

**Table 3.** Clinical staffing patterns in health centres and health posts, by country (number/proportion and 95% CI)

	Kenya	Madagascar	Malawi	Mozambique	Niger	Nigeria	Sierra Leone	Tanzania	Togo	Uganda	All
	*n* = 2753	*n* = 407	*n* = 1005	*n* = 157	*n* = 239	*n* = 1974	*n* = 506	*n* = 353	*n* = 164	*n* = 385	*n* = 7943
Average number of clinical staff in health centres and health posts	3.35 (3.15–3.55)	2.17 (1.92–2.41)	3.37 (3.12–3.62)	5.87 (4.33–7.4)	1.38 (0.95–1.82)	0.58 (0.5–0.67)	1.20 (0.95–1.44)	4.36 (3.71–5)	2.60 (1.88–3.33)	3.42 (3.05–3.79)	2.83 (1.69–3.97)
Proportion of health centres and health posts with one or fewer clinical staff	33.1 (31.3–35.0)	50.0 (40.6–59.4)	31.9 (29.1–34.8)	37.6 (30.0–45.2)	79.2 (73.1–85.3)	88.1 (86.4–89.8)	77.5 (73.3–81.6)	22.0 (16.1–28)	48.2 (32.5–63.8)	28.7 (23.2–34.3)	49.6 (32.7–66.6)
Proportion of health centres and health posts with two or fewer clinical staff	62.1 (60.1–64)	82.4 (76.8–88.1)	53.6 (50.5–56.7)	58.6 (50.9–66.3)	88.0 (83.5–92.4)	93.5 (92.2–94.8)	87.4 (83.9–90.9)	47.6 (39.9–55.4)	67.0 (52.2–81.8)	54.5 (48.2–60.8)	69.5 (57.5–81.5)

When we examined the distribution of health worker cadres by facility type, we found a wide variation in the number of health workers deployed to facilities by country (Table 2). In some countries such as Kenya and Uganda, health worker cadres showed a similar distribution across facility types with the nurse/midwife being the primary cadre, followed by a substantial proportion of non-clinical/other workers and supported by a smaller proportion of doctors/clinical officers. In other countries such as Malawi, Niger and Nigeria, we found that health posts were primarily staffed by non-clinical/other health workers, health centres were primarily staffed by nurses/midwives and non-clinical/other health workers, and hospitals were primarily staffed by nurses/midwives supported by doctors/clinical officers and non-clinical/other health workers. Across all countries combined, we found that hospitals had the largest proportion of doctors/clinical officers (19.4% compared with 14% for health centres and 7.5% for health posts) and the largest proportion of nurse/midwives (47.5% compared with 40.9% for health centres and 33.3% for health posts).

When we examined staffing patterns (Table 3), we found that, on average, health centres and health posts had 2.8 clinical staff deployed to facilities, but many facilities had one or no clinical staff. In Madagascar, Niger, Nigeria and Sierra Leone, more than half of health centres and health posts had one or no clinical staff deployed to facilities.^12^ In addition, nearly 70% of health centres and health posts across all countries had two or fewer clinical staff deployed to facilities. This lack of clinical staff deployed to facilities may not be indicative of no patient care provision at these facilities; however, it may suggest that patient care is being provided by under-qualified individuals.^13^

These average levels of clinical staff are far below staffing norms. Supplementary Table S5 shows staffing norms—by cadre and health facility type—for each country. Supplementary Table S6 shows the average staffing norms across cadres and types, compared with the average staffing levels from Table 3. In every case, the staff deployed to facilities is less than half that listed in country staffing plans.

### Are health workers present? Health worker absence levels, effective staffing patterns and correlates

We first looked at the overall health worker absences from facilities to understand the extent to which health workers who are expected to be present at health facilities are not present for work (Supplementary Table S7). We then explored the relationship between health worker absence from the facility and facility and health worker characteristics to further understand if health workers with certain characteristics were more likely to be absent (Table 4). Finally, we examined staffing patterns adjusted for health worker absence from facilities to see the impact of health worker absence on the proportion of facilities with limited clinical staff present (Supplementary Table S10).

**Table 4.** Logistic regression of health worker absence from facilities on facility and health worker characteristics

	Odds ratio	95% CI	*P*-value
Facility type (REF = hospital)			
Health centre	0.992	(0.910–1.082)	0.863
Health post	0.786	(0.715–0.864)	<0.001*
Managing authority (REF = private/NGO)			
Public	1.399	(1.295–1.512)	<0.001*
Urban/rural (REF = urban)			
Rural	1.043	(0.972–1.118)	0.245
Health worker cadre (REF = doctor/clinical officer)			
Nurse/midwife	0.936	(0.865–1.012)	0.095
Other workers	0.917	(0.846–0.995)	0.037*
Health worker sex (REF = female)			
Male	0.966	(0.917–1.018)	0.194
Health worker age (REF = <30 years)			
30–40 years	1.147	(1.079–1.219)	<0.001*
40–50 years	1.090	(1.015–1.171)	0.018*
>50 years	1.113	(1.028–1.206)	0.008*
Health worker education (REF = primary)			
Secondary	0.943	(0.820–1.084)	0.407
Post-secondary	0.868	(0.748–1.007)	0.062
Country (REF = Kenya)			
Madagascar	0.395	(0.341–0.457)	<0.001*
Malawi	0.372	(0.328–0.421)	<0.001*
Mozambique	0.235	(0.195–0.284)	<0.001*
Niger	0.387	(0.320–0.468)	<0.001*
Nigeria	0.461	(0.413–0.514)	<0.001*
Sierra Leone	0.652	(0.563–0.755)	<0.001*
Tanzania	0.178	(0.151–0.209)	<0.001*
Togo	0.516	(0.430–0.620)	<0.001*
Equipment availability	1.000	(0.999–1.000)	0.198
Infrastructure availability	1.000	(0.999–1.000)	0.529
(Intercept)	1.089	(0.876–1.355)	0.442

*denotes statistical significance at the *p*<0.05 level, indicating that the observed results are unlikely to have occurred by chance.

**Figure 1.**
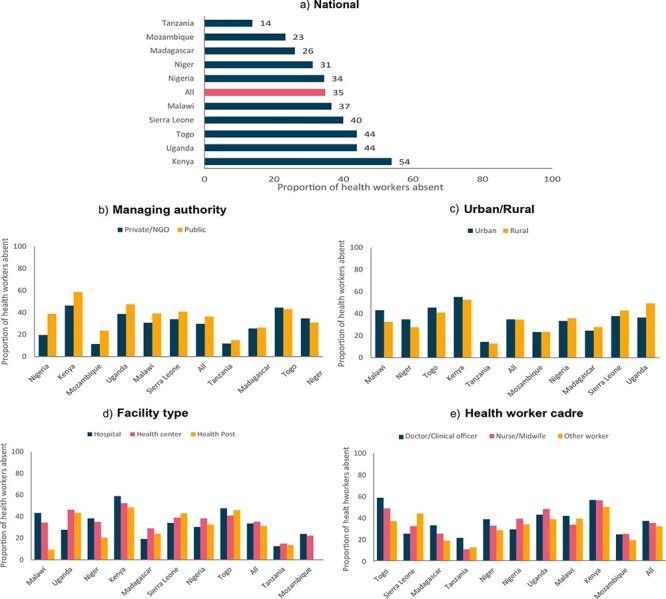
National and sub-national variation in health worker absence from facilities

We found that the rate of overall health worker absence from facilities was, on average, 34.7% (CI: 26.3–43%). On average, across all countries, health worker absence from facilities was similarly high across all types of health facilities (hospital 33.6%, health centre 35.2% and health post 31.2%), all cadres of health workers (doctors/clinical officers 36.8%, nurses/midwives 34.8% and other workers 31.8%) and in both urban and rural facilities (urban 34.8% and rural 34.6%). Across all countries, on average, a patient was more likely to find a health worker in a private/NGO facility compared with a public facility, but health worker absence from facilities was high across facilities of all managing authorities (public 36.4% and private/NGO 29.7%) (Supplementary Table S7 and Figure 1).

However, there was substantial variation in health worker absence from facilities both within and between countries. The proportion of health workers absent from facilities ranged from 53.8% in Kenya to 13.8% in Tanzania. Health worker absence from facilities was statistically significantly higher in public facilities than in private facilities in three countries (Nigeria 19 percentage points (pp) higher, Kenya 12 pp higher and Malawi 9 pp higher). While overall, health worker absence from facilities was similar in urban and rural areas, this varied by country, with about half of countries having higher health worker absence from facilities in urban areas and half of countries having higher health worker absence from facilities in rural areas, but these differences were generally not significant. No strong pattern emerged across countries related to health worker absence from facilities by facility type or health worker cadre. We did not see a single facility type or health worker cadre have consistently higher health worker absence from facilities compared with other groups across countries (Supplementary Table S7 and Figure 1).

Results from the logistic regression of health worker absence from facilities on facility and health worker characteristics indicate that both facility and health worker characteristics are associated with health worker absence from facilities (Table 4). The facility-level covariates that were statistically significantly associated with health worker absence from facilities include facility type and managing authority. Health worker absence from facilities was higher for health workers at public facilities (β = 1.399, *P*-value < 0.001) compared with private facilities and lower for health workers at health posts (β = 0.786, *P*-value < 0.001) as compared with health workers at hospitals. The health worker–level covariates that were statistically significantly associated with health worker absence from facilities include cadre and age. Health worker absence from facilities was lower for other workers (β = 0.917, *P*-value = 0.037) as compared with doctors while health worker absence from facilities was higher for health workers of all age groups compared with health workers <30 years. Our findings demonstrate that health worker absence from facilities was lowest at the smallest health facilities.

Further analysis into the reasons for health worker absences revealed that there was very little unauthorized absence. Across all countries, the rate of unauthorized health worker absence from facilities was, on average, 3.9% (CI: 1.8–5.9%) and no country had an unauthorized health worker absence rate above 9% (Supplementary Table S8). There were a broad range of reasons given for health worker absences including authorized absence, official mission, outreach, or fieldwork, sick or maternity leave, training/seminar/meeting and unauthorized absence (Supplementary Table S9). Our findings indicate that high health worker absence from facilities is not indicative of a moral failure on the part of health workers but instead is a characteristic of the health system.

**Figure 2.**
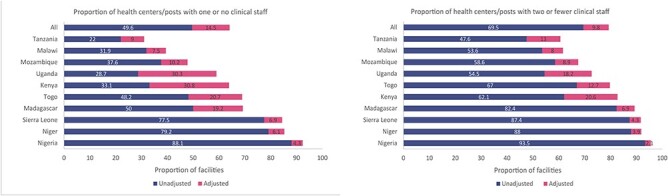
Clinical staffing patterns by country, unadjusted and adjusted for health worker absence from facilities at primary care level

We went on to examine staffing patterns adjusted for health worker absence from facilities to see the impact of health worker absence from facilities on the proportion of facilities with limited clinical staff present (Supplementary Table S10 and Figure 2). Due to the different distribution of primary and secondary facilities across countries, this analysis focused on primary care facilities only. After adjusting for health worker absence from facilities, the staffing patterns in health centres and health posts were much worse. Across all countries, the proportion of health centres and health posts with one or no clinical staff increased from 49.6% to 64.1% and the proportion of health centres and health posts with two or fewer clinical staff increased from 69.5% to 79.3% after adjusting for health worker absence from facilities. In addition, more than half of health centres and health posts in 7 out of 10 countries had one or no clinical staff after adjusting for health worker absence from facilities. The countries in which adjusting for health worker absence from facilities had the largest impact on the proportion of health centres and health posts with one or no clinical staff include Kenya (33.1% to 63.9%), Togo (48.2% to 68.9%) and Uganda (28.7% to 59.0%).

### How heavy are health workers’ caseloads?

In order to understand staffing needs beyond the proportion of facilities with only one or two clinical staff, we first looked at health worker caseload to understand how many clients health workers are seeing per day (Table 5). We then examined the proportion of facilities with low and high caseload to understand how many facilities are potentially not maximizing productivity (Table 6). Finally, we explored the relationship between caseload and facility characteristics to further understand if facilities with certain characteristics were more likely to have higher caseloads (Table 7). Malawi was excluded from the caseload analysis due to potential discrepancies in the collection of data on outpatient visits. The caseload analysis including Malawi is provided in the supplementary materials (Supplementary Tables S11, S12, S13 and Supplementary Figures S1a and S1b).

**Table 5.** Average health worker caseload per facility, by country (number and 95% CI)

	Kenya	Madagascar	Mozambique	Niger	Nigeria	Sierra Leone	Tanzania	Togo	Uganda	All
National	23.1 (21.9–24.2)	6.4 (5.0–7.7)	23.2 (20.0–26.5)	10.9 (8.6–13.2)	2.8 (2.5–3.2)	11.3 (10.2–12.5)	11.7 (9.6–13.8)	6.3 (4.8–7.9)	17.0 (14.2–19.7)	12.5 (6.9–18.1)
Facility type										
Hospital	24.6 (19.9–29.2)	5.5 (3.1–7.8)	16.7 (7.9–25.5)	1.1 (0.6–1.7)	4.1 (2.9–5.4)	8.0 (6.3–9.8)	33.5 (5.5–61.4)	6.6 (4.0–9.3)	16.6 (2.9–30.2)	13.0 (4.7–21.2)
Health centre	25.0 (22.4–27.5)	7.7 (5.5–9.9)	24.9 (21.5–28.3)	21.6 (17.1–26.2)	2.7 (2.3–3.0)	11.3 (8.7–13.9)	14.2 (7.6–20.9)	7.6 (4.5–10.6)	17.1 (11.8–22.4)	14.7 (8.5–20.9)
Health post	22.2 (21–23.5)	4.0 (2.8–5.2)	NA	6.1 (4.5–7.6)	2.0 (1.7–2.3)	11.5 (10.1–12.9)	10.4 (8.5–12.4)	5.8 (4.1–7.6)	16.9 (13.7–20.2)	9.9 (4.1–15.6)
Managing authority										
Private/NGO	15.0 (13.5–16.6)	11.8 (9.8–13.7)	12.5 (0.0–29.0)	5.0 (2.6–7.4)	3.2 (1.9–4.6)	9.2 (7.1–11.3)	11.3 (7.0–15.6)	6.8 (4.3–9.4)	7.0 (4.7–9.4)	9.1 (6.1–12.1)
Public	30.4 (28.9–32.0)	5.1 (3.6–6.6)	23.4 (20.0–26.7)	11.2 (8.7–13.6)	2.7 (2.5–3.0)	11.5 (10.2–12.8)	11.9 (9.4–14.3)	6.0 (4.0–8.0)	26.1 (22.0–30.3)	14.3 (6.6–21.9)
Urban/rural										
Urban	20.1 (17.5–22.6)	10.5 (8.7–12.3)	19.6 (5.6–33.6)	8.1 (4.5–11.7)	3.2 (2.5–3.9)	10.5 (8.5–12.5)	15.3 (9.7–20.9)	6.6 (4.3–9.0)	10.1 (5.7–14.6)	11.6 (7.2–16.0)
Rural	24.6 (23.4–25.7)	4.9 (3.3–6.6)	23.7 (20.5–26.9)	11.2 (8.7–13.8)	2.6 (2.3–2.9)	11.7 (10.2–13.2)	10.1 (8.4–11.8)	6.1 (4.0–8.2)	20.2 (16.8–23.5)	12.8 (6.5–19.1)

**Table 6.** Proportion of facilities with low and high caseload, by country (% and 95% CI)

	Kenya	Madagascar	Mozambique	Niger	Nigeria	Sierra Leone	Tanzania	Togo	Uganda	All
Proportion of facilities with a caseload of <5										
Private/NGO	31.7 (28.7–34.6)	30.7 (19.7–41.8)	50.0 (0 0.0–119.9)	70.5 (52.6–88.3)	83.1 (76.2–89.9)	32.5 (16.8–48.1)	40.5 (24.7–56.4)	53.2 (23.3–83.2)	64.1 (54.1–74.2)	50.7 (36.3–65.1)
Public	5.0 (3.8–6.1)	73.1 (64.8–81.5)	13.6 (8.6–18.6)	40.7 (30.3–51.1)	86.5 (84.7–88.3)	32.3 (27.6–37.1)	35.6 (26.7–44.5)	63.9 (50.2–77.7)	3.4 (0.5–6.3)	39.3 (16.3–62.3)
Total	17.8 (16.1–19.4)	65.2 (57.0–73.4)	14.0 (9.0–19.0)	41.8 (31.8–51.8)	85.9 (84.0–87.8)	32.4 (27.8–36.9)	37.0 (29.2–44.7)	59.5 (44.9–74.1)	32.5 (25.6–39.4)	42.9 (25.0–60.8)
Proportion of facilities with a caseload of ≥30										
Private/NGO	12.8 (10.5–15.1)	9.1 (4.3–13.9)	0.0 (0.0–0.0)	0.0 (0.0–0.0)	1.9 (0.0–4.4)	0.0 (0.0–0.0)	3.7 (0.1–7.3)	0.0 (0.0–0.0)	4.7 (0.7–8.7)	3.6 (0.0–7.1)
Public	36.1 (33.4–38.7)	1.2 (0.0–2.3)	27.2 (20.7–33.7)	10.7 (4.1–17.2)	0.8 (0.3–1.3)	6.2 (4.1–8.4)	6.5 (3.8–9.2)	3.8 (0.0–11.2)	29.8 (22.4–37.2)	13.6 (3.1–24.0)
Total	24.9 (23.1–26.8)	2.6 (1.3–4.0)	26.9 (20.5–33.3)	10.3 (4.0–16.5)	1.0 (0.4–1.6)	5.7 (3.7–7.7)	5.7 (3.5–7.9)	2.2 (0.0–6.6)	17.8 (13.2–22.3)	10.8 (3.1–18.5)

**Table 7.** Linear regression of caseload on facility characteristics

	Model 1—facility characteristics on caseload	Model 2—Model 1 + readiness	Model 3—Model 1 + readiness + absenteeism	Model 4—Model 1 + readiness + absenteeism + competency
Country (REF = Kenya)				
Madagascar	−15.1 ***	−14.2 ***	−10.5 ***	−9.1 ***
Mozambique	−5.7 **	−5.1 **	0.0	2.2
Niger	−16.8 ***	−15.9 ***	−12.0 ***	−9.5 ***
Nigeria	−25.3 ***	−24.0 ***	−21.1 ***	−18.3 ***
Sierra Leone	−14.0 ***	−13.5 ***	−11.9 ***	−10.5 ***
Tanzania	−10.4 ***	−10.3 ***	−5.9 ***	−5.3 ***
Togo	−19.5 ***	−19.3 ***	−17.7 ***	−16.3 ***
Uganda	−4.7 ***	−4.2 ***	−4.0 ***	−2.0
Facility type (REF = hospital)				
Health centre	−1.4	−1.1	−1.0	−0.4
Health post	−3.2 ***	−2.4 **	−0.4	0.4
Managing authority (REF = private/NGO)				
Public	10.9 ***	11.2 ***	9.7 ***	9.4 ***
Urban/rural (REF = urban)				
Rural	−0.9	−0.6	−0.2	−0.2
Equipment availability		0.0 ***	0.0 **	0.01 **
Infrastructure availability		0.0 **	0.0 *	0.01
Health worker absence from facilities			0.2 ***	0.2 ***
Provider competency				0.1 ***
(Intercept)	20.1 ***	16.5 ***	9.4 ***	5.4 ***
*R*-squared	0.2262	0.2284	0.2834	0.2892

Significance codes: ‘***’ 0.001, ‘**’ 0.01, ‘*’ 0.05.

Note: *n* = 6763; 1047 facilities excluded due to missing data on caseload. Reasons for missing caseload data include the following:

The facility was missing data on the number of staff.

The facility was missing data on the number of outpatient visits.

The caseload value was set to missing/omitted because it was greater than 200, which was deemed to be unrealistically high.

When examining health worker caseload, we found that, on average, a health worker attended to 12.5 outpatients per day (CI: 6.9–18.1). On average, across all countries, caseload was similar across all types of health facilities (hospital 13, health centre 14.7 and health post 9.9), in both private/NGO and public facilities (private/NGO 9.1 and public 14.3) and in both urban and rural facilities (urban 11.6 and rural 12.8). However, health worker caseload varied substantially both across and within countries. Health worker caseload ranged from a low of 2.8 outpatients per day (CI: 2.5–3.2) in Nigeria to a high of 23.2 outpatients per day in Mozambique (CI: 20–26.5) and 23.1 outpatients per day in Kenya (CI: 21.9–24.2). While there is no global benchmark for caseload, some basic assumptions can be made to better contextualize caseload values. Assuming a health worker saw one patient every half hour over the course of an eight-hour shift, this would be a caseload of 16 outpatients per day. Six out of nine countries (Madagascar, Niger, Nigeria, Sierra Leone, Tanzania and Togo) had an average caseload lower than 16 outpatients per day. Caseload was statistically significantly higher in public facilities than in private facilities in three countries (Kenya, Niger and Uganda), while one country, Madagascar, had diverging findings with caseload statistically significantly higher in the private sector (7 pp). Only three countries had a statistically significant difference between caseload in urban and rural facilities, with caseload being higher in urban facilities in Madagascar and higher in rural facilities in Kenya and Uganda. No strong pattern emerged across countries related to caseload by facility type. We did not see a single facility type have consistently higher caseload compared with other groups across countries (Table 5).

**Figure 3.**
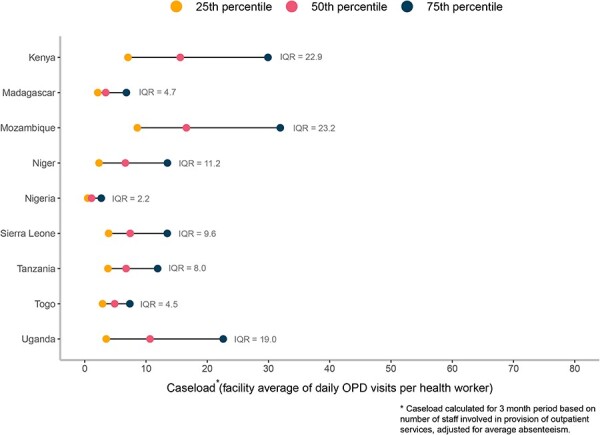
Caseload distribution, by country

We then examined the proportion of facilities with very low and very high caseload, both which may be suggestive of health system inefficiencies. The distribution of caseload by country shows that in countries such as Mozambique, Kenya and Uganda, there were some facilities with a much higher caseload than other facilities, while in other countries such as Nigeria, Togo and Madagascar, caseload was similar across all facilities. The distribution of caseload also points to several countries in which most facilities had a rather low caseload (Figure 3). We assessed the proportion of facilities with a caseload of less than 5 outpatients per day (low caseload) and the proportion of facilities with a caseload greater than or equal to 30 outpatients per day (high caseload). Across all countries, 42.9% of facilities had a caseload of less than five outpatients per day. More than half of facilities in Madagascar, Nigeria and Togo had a caseload of less than five outpatients per day. In three countries (Kenya, Niger and Uganda), the proportion of facilities with a caseload of less than five outpatients per day was substantially higher for the private/NGO sector as compared with the public sector, while in one country, Madagascar, the proportion of facilities with a caseload of less than five outpatients per day was substantially higher for the public sector as compared with the private sector.

Across all countries, 10.8% of facilities had a caseload of 30 or more outpatients per day. Approximately one-quarter of facilities in Kenya and Mozambique had a caseload of 30 or more outpatients per day, while 10% or less of facilities in Madagascar, Niger, Nigeria, Tanzania and Togo had a caseload of 30 or more. In five countries (Kenya, Mozambique, Niger, Sierra Leone and Uganda), the proportion of facilities with a caseload of 30 or more outpatients per day was substantially higher for the public sector as compared with the private (Table 6).

Finally, we explored associations between caseload and facility characteristics to further understand if facilities with certain characteristics were more likely to have higher or lower caseloads. Results from a linear regression of caseload on facility characteristics, readiness, health worker absence from facilities and competency indicate that public facilities have much higher caseloads than private/NGO facilities (β = 9.371, *P*-value < 0.001). In addition, caseload is modestly higher when both health worker absence from facilities (β = 0.189, *P*-value < 0.001) and competency are higher (β = 0.073, *P*-value < 0.001). There was no difference in caseload between urban and rural facilities or between health centres and hospitals after controlling for other facility characteristics. In addition, increases in readiness were not all associated with increased caseload, with only increases in equipment availability being associated with a small increase in caseload (β = 0.011, *P*-value < 0.01) (Table 7).

### How competent are health workers?

To understand health worker competency, we first looked at a measure of diagnostic and treatment accuracy. As diagnostic accuracy and treatment accuracy are highly correlated, we present here the combined indicator for both diagnostic and treatment accuracy (Table 8). Detailed results for diagnostic accuracy and treatment accuracy individually can be found in Supplementary Tables S14 and S15, respectively. We then examined the proportion of health workers with a diagnostic and treatment accuracy score of 50% or less, 20% or less and equal to 0% (Table 9). Finally, we explored the relationship between diagnostic and treatment accuracy and facility and health worker characteristics (Table 10).

**Table 8.** Health worker diagnostic and treatment accuracy, by country (% and 95% CI)

	Kenya	Madagascar	Malawi	Mozambique	Niger	Nigeria	Sierra Leone	Tanzania	Togo	Uganda	All
National	74.4 (73.1–75.6)	55.9 (51.4–60.3)	77.1 (75.3–78.8)	46.3 (43.2–49.4)	41.0 (38.6–43.5)	35.0 (32.9–37.1)	57.4 (55.6–59.2)	70.1 (66.9–73.3)	55.9 (49.5–62.4)	47.9 (43.7–52.2)	56.1 (46.0–66.2)
Facility type											
Hospital	78.9 (75.1–82.6)	61.6 (50.2–73.0)	79.2 (76.7–81.7)	49.5 (45.3–53.7)	43.8 (37.7–49.8)	47.7 (43.9–51.5)	66.4 (60.3–72.5)	75.8 (71.1–80.5)	56.5 (48.4–64.6)	71.4 (67.2–75.6)	63.1 (53.6–72.6)
Health centre	74.4 (72.4–76.4)	56.8 (51.1–62.5)	74.0 (71.8–76.2)	39.5 (36.2–42.8)	41.1 (37.3–44.9)	24.4 (23.0–25.8)	59.6 (56.1–63.1)	74.6 (69.4–79.7)	65.1 (56.7–73.5)	56.9 (52.4–61.5)	56.7 (44.5–68.8)
Health post	71.8 (70.7–72.8)	49.6 (43.1–56.1)	64.2 (56.4–71.9)	NA	39.6 (35.9–43.3)	21.4 (19.3–23.5)	55.2 (53.0–57.4)	52.1 (48.3–56.0)	48.0 (39.5–56.5)	28.2 (24.9–31.6)	47.8 (35.4–60.2)
Managing authority											
Private/NGO	68.7 (67.0–70.3)	49.4 (40.5–58.2)	75.3 (72.6–78.0)	50.8 (40.4–61.2)	44.4 (36.9–51.9)	46.0 (39.7–52.3)	62.3 (57.2–67.4)	69.8 (65.0–74.6)	57.8 (45.6–70.0)	47.3 (39.4–55.2)	57.2 (49.1–65.2)
Public	77.6 (75.9–79.3)	58.0 (52.9–63.1)	77.7 (75.5–80.0)	46.2 (43.1–49.4)	40.8 (38.2–43.4)	32.6 (30.7–34.4)	56.7 (54.8–58.6)	70.3 (66.2–74.3)	54.3 (47.6–61.0)	48.3 (43.4–53.3)	56.3 (45.4–67.1)
Urban/Rural											
Urban	73.6 (70.7–76.5)	51.0 (45.0–57.0)	78.8 (76.2–81.5)	52.7 (45.4–60.0)	40.0 (36.1–43.9)	38.7 (35.7–41.8)	60.6 (57.9–63.4)	75.3 (72.1–78.6)	58.8 (49.6–68.0)	58.3 (52.0–64.6)	58.8 (48.8–68.8)
Rural	74.8 (73.9–75.8)	58.6 (52.5–64.6)	74.7 (72.6–76.8)	43.5 (40.7–46.3)	42.0 (38.8–45.2)	30.1 (27.3–32.9)	54.8 (52.4–57.2)	56.3 (51.7–61.0)	51.3 (41.7–60.8)	39.9 (36.5–43.3)	52.6 (42.2–63.0)
Health worker cadre											
Doctor/clinical officer	77.2 (75.0–79.4)	60.0. (54.2–65.8)	79.5 (76.6–82.4)	51.9 (47.4–56.5)	46.3 (30.8–61.8)	54.2 (48.8–59.7)	67.5 (56.0–79.0)	73.9 (70.7–77.1)	66.3 (57.3–75.3)	64.6 (59.3–70.0)	64.2 (56.2–72.1)
Nurse/midwife	71.9 (70.8–73.0)	53.0 (46.6–59.4)	76.4 (74.1–78.6)	38.6 (34.5–42.7)	41.0 (37.6–44.5)	40.5 (36.4–44.6)	59.6 (56.2–62.9)	46.8 (39.9–53.8)	54.0 (46.0–62.0)	39.5 (34.5–44.5)	52.1 (42.4–61.9)
Other workers	50.8 (43.8–57.7)	40.9 (26.5–55.3)	52.9 (43.1–62.7)	41.2 (34.7–47.7)	40.2 (36.7–43.6)	22.1 (20.7–23.5)	56.0 (53.9–58.2)	39.5 (31.1–47.9)	31.2 (22.6–39.9)	23.5 (19.2–27.8)	39.8 (31.5–48.1)

**Table 9.** Proportion of health workers with low competency, by country (% and 95% CI)

	Kenya	Madagascar	Malawi	Mozambique	Niger	Nigeria	Sierra Leone	Tanzania	Togo	Uganda	All
A. Proportion of health workers with diagnostic and treatment accuracy 50% or less											
National	57.0 (53.9–60.1)	89.3 (84.4–94.2)	27.7 (22.4–33.0)	74.1 (68.2–80.0)	91.9 (88.9–94.9)	85.8 (82.7–88.9)	76.8 (73.5–80.2)	30.2 (20.5–40.0)	70.4 (58.1–82.8)	75.8 (67.2–84.3)	67.9 (51.6–84.2)
Health worker cadre											
Doctor/clinical officer	47.2 (42.1–52.3)	85.4 (77.7–93.1)	20.4 (10.7–30.1)	64.1 (54.9–73.4)	75.6 (57.7–93.6)	70.5 (61.0–79.9)	52.4 (30.2–74.5)	22.8 (12.3–33.3)	41.0 (20.9–61.1)	58.8 (40.7–77)	53.8 (38.4–69.2)
Nurse/midwife	67.4 (65.2–69.6)	92.1 (85.7–98.6)	32.0 (26.5–37.6)	87.7 (82.1–93.3)	92.2 (88.0–96.5)	85.9 (80.7–91.1)	76.5 (70.5–82.5)	80.9 (70.9–90.8)	79.0 (66.1–92.0)	85.2 (75.9–94.5)	77.9 (65.2–90.6)
Other workers	83.4 (74.1–92.8)	100.0 (100.0–100.0)	58.0 (41.3–74.7)	83.5 (72.7–94.2)	94.1 (90.0–98.2)	94.4 (92.8–95.9)	77.7 (73.6–81.9)	80.8 (68.0–93.6)	93.7 (81.2–100.0)	98.7 (96.8–100.0)	86.4 (77.4–95.5)
B. Proportion of health workers with diagnostic and treatment accuracy 20% or less											
National	6.3 (4.2–8.4)	24.2 (16.2–32.2)	7.8 (5.8–9.8)	44.3 (38.4–50.2)	71.1 (65.7–76.6)	66.6 (62.8–70.5)	49.6 (45.5–53.7)	13.3 (8.3–18.4)	32.9 (22.2–43.6)	42.5 (34.5–50.5)	35.9 (19.3–52.4)
Health worker cadre											
Doctor/clinical officer	5.2 (1.3–9.0)	10.7 (4.9–16.5)	2.6 (1.0–4.2)	34.3 (25.9–42.8)	32.6 (8.9–56.3)	33.6 (23.2–43.9)	36.0 (11.2–60.8)	6.9 (2.3–11.5)	29.3 (12.1–46.6)	11.9 (5.7–18.0)	20.3 (10.4–30.3)
Nurse/midwife	6.8 (5.6–8.1)	34.6 (21.8–47.3)	10.4 (7.3–13.5)	57.1 (48.9–65.4)	68.1 (60.1–76.1)	64.0 (57.9–70.2)	41.6 (34.3–48.8)	53.4 (38.3–68.6)	32.5 (18.9–46.1)	59.9 (51.1–68.6)	42.8 (27.4–58.3)
Other workers	31.2 (21.3–41.2)	48.1 (18.7–77.5)	38.2 (23.5–53.0)	55.3 (40.3–70.2)	81.6 (75.0–88.1)	86.2 (84.1–88.2)	53.6 (48.6–58.6)	63.9 (48.3–79.6)	56.0 (17.6–94.4)	82.8 (74.1–91.5)	59.7 (46.2–73.2)
C. Proportion of health workers with diagnostic and treatment accuracy equal to 0%											
National	6.3 (4.2–8.4)	24.2 (16.2–32.2)	2.7 (1.8–3.7)	14.4 (10.4–18.4)	39.1 (33.1–45.2)	46.7 (43.3–50.2)	22.4 (18.9–26.0)	4.8 (2.8–6.9)	13.5 (7.3–19.8)	24.8 (19.5–30.1)	19.9 (9.5–30.4)
Health worker cadre											
Doctor/clinical officer	5.2 (1.3–9)	10.7 (4.9–16.5)	0.4 (0.1–0.7)	9.1 (3.6–14.6)	24.3 (0.1–48.6)	19.9 (10–29.7)	5.5 (0–13.8)	1.5 (0.5–2.5)	12.3 (0.1–24.4)	4.1 (1.1–7.1)	9.3 (3.7–14.9)
Nurse/midwife	6.8 (5.6–8.1)	34.6 (21.8–47.3)	2.9 (1.4–4.4)	22.0 (14.9–29.1)	30.4 (22.8–38.0)	36.8 (31.4–42.2)	17.5 (11.8–23.2)	27.1 (13.0–41.1)	12.6 (5.1–20.1)	32.5 (25.5–39.5)	22.3 (13.8–30.9)
Other workers	31.2 (21.3–41.2)	48.1 (18.7–77.5)	33.0 (19.2–46.8)	18.8 (9.3–28.2)	53.6 (44.3–62.9)	65.5 (63.2–67.9)	25.2 (20.7–29.7)	27.8 (12.7–42.8)	32.6 (5.7–59.6)	62.6 (52.8–72.3)	39.8 (28.1–51.5)

**Table 10.** Linear regression of diagnostic and treatment accuracy on facility and health worker characteristics

	Estimate	Std. error	*P*-value
Facility type (REF = hospital)			
Health centre	−5.193	0.835	<0.001*
Health post	−6.977	0.893	<0.001*
Managing authority (REF = private/ NGO)			
Public	4.901	0.680	<0.001*
Urban/rural (REF = Urban)			
Rural	1.488	0.569	0.009*
Health worker cadre (REF = doctor/clinical officer)			
Nurse/midwife	−7.813	0.645	<0.001*
Other workers	−16.207	0.942	<0.001*
Health worker sex (REF = female)			
Male	4.579	0.445	<0.001*
Health worker age (REF = <30 years)			
30–40 years	0.759	0.535	0.156
40–50 years	−0.077	0.603	0.898
>50 years	−1.833	0.678	0.007*
Health worker education (REF = primary)			
Secondary	1.952	1.350	0.148
Post-secondary	5.622	1.436	<0.001*
Country (REF = Kenya)			
Madagascar	−20.422	1.165	<0.001*
Malawi	3.915	1.111	<0.001*
Mozambique	−25.324	1.245	<0.001*
Niger	−27.156	1.200	<0.001*
Nigeria	−27.330	1.053	<0.001*
Sierra Leone	−9.043	1.202	<0.001*
Tanzania	−5.886	1.475	<0.001*
Togo	−18.479	1.569	<0.001*
Equipment availability	0.007	0.006	0.203
Infrastructure availability	0.026	0.006	<0.001*
(Intercept)	49.581	1.935	<0.001*
*R*-squared	0.4202		

*denotes statistical significance at the *p*<0.05 level, indicating that the observed results are unlikely to have occurred by chance.

When examining health worker competency, we found that diagnostic and treatment accuracy was, on average, 56.1% (CI: 46–66.2%), meaning that, on average, health workers gave the correct diagnosis and treatment for just over half of cases. On average, across all countries, diagnostic and treatment accuracy was similar in both urban and rural facilities (58.8% and 52.6%) and public and private/NGO facilities (56.3% and 57.2%). However, it differed by facility type and health worker cadre and a decreasing trend in health worker competency was seen as the facility level decreased from hospital (63.1%) to health centre (56.7%) to health post (47.8%) and as the health worker level of training decreased from doctor/clinical officer (64.2%) to nurse/midwife (52.1%) to other workers (39.8%).

Diagnostic and treatment accuracy varied by country and was highest in Malawi (77.1%, CI: 75.3–78.8%) and lowest in Nigeria (35%, CI: 32.9–37.1%). The range of health worker competency between health worker cadres varied substantially by country. The difference in diagnostic and treatment accuracy between doctors/clinical officers and nurses/midwifes ranged from a 27-pp difference in Tanzania to a 3-pp difference in Malawi, while the difference in diagnostic and treatment accuracy between nurses/midwives and other workers ranged from a 24-pp difference in Malawi to a 1-pp difference in Niger (Table 8).

**Figure 4.**
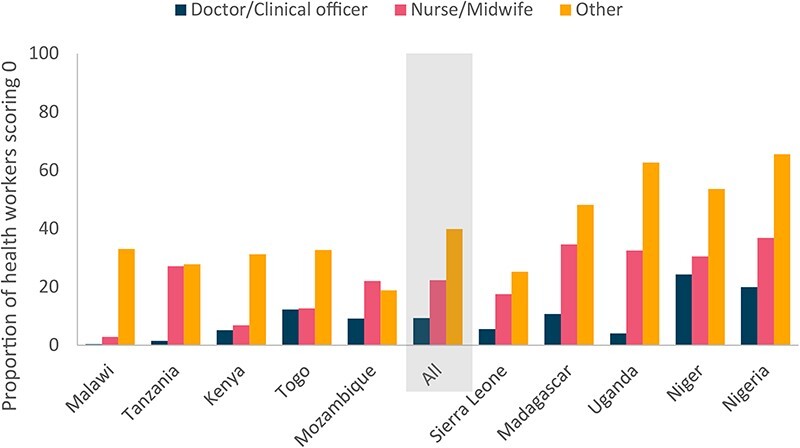
Proportion of health workers with a score of zero on a series of vignettes

We also examined the proportion of health workers with a diagnostic and treatment accuracy score of 50% or less, 20% or less and equal to 0%. Across all countries, 67.9% of health workers (CI: 51.6–84.2%) had a diagnostic and treatment accuracy score of 50% or less, 35.9% of health workers (CI: 19.3–52.4%) had a diagnostic and treatment accuracy score of 20% or less, and 19.9% of health workers (CI: 9.5–30.4%) had a diagnostic and treatment accuracy score equal to 0%. The proportion of health workers with a competency score of 0 was, on average, 9.3% for doctors/clinical officers, 22.3% for nurses/midwives and 39.8% for other workers (Table 9). However, the proportion of health workers with a competency score of 0 varied by country, with countries such as Madagascar, Niger and Nigeria, having >10% of doctors/clinical officers and >30% of nurses/midwives with a competency score of 0 (Figure 4).

Finally, we explored the relationship between diagnostic and treatment accuracy and facility and health worker characteristics. Health worker diagnostic and treatment accuracy was lower for health workers at health centres (β = −5.193, *P*-value < 0.001) and health posts (β = −6.977, *P*-value < 0.001) as compared with health workers at hospitals. However, health worker diagnostic and treatment accuracy was higher for health workers at rural facilities (β = 1.488, *P*-value = 0.009) compared with urban facilities, for health workers at public facilities (β = 4.901, *P*-value < 0.001) compared with private/NGO facilities and for facilities with higher levels of infrastructure availability (β = 0.026, *P*-value = 0.006). Equipment availability was the only facility-level covariate not statistically significantly associated with health worker diagnostic and treatment accuracy, thus not supporting the idea that more competent health workers choose to work in better equipped facilities.

The health worker–level covariates that were statistically significantly associated with diagnostic and treatment accuracy include cadre, age, sex and education. Health worker diagnostic and treatment accuracy was lower for nurses (β = −7.813, *P*-value < 0.001) and other workers (β = −16.207, *P*-value < 0.001) as compared with doctors as well as for health workers over the age of 50 years (β = −1.8313, *P*-value = 0.007) as compared with those under the age of 30 years. In addition, health worker diagnostic and treatment accuracy was higher for male health workers (β = 4.579, *P*-value < 0.001) (Table 10).^14^

Our findings demonstrate that health workers in health centres and health posts have lower competency than those in hospitals, which means the least qualified health workers are at the frontline of primary health care. In addition, the public sector draws competent health workers as evidenced by the higher competency of public sector health workers as compared with private sector health workers. Finally, rural health workers have slightly higher competency as do men and younger health workers.

## Discussion

### Key findings

This study utilized SDI data to explore health workforce challenges across and within 10 countries in SSA. We found that, on average, health centres and health posts have between 2.5 and 3 clinical health workers—well below the expected staffing norms—but that there are many health centres or health posts with one or no clinical health workers. Thus, even if health facilities are reasonably staffed on average, this masks significant shortages within countries. We also found high levels of health worker absence from facilities across all types of health facilities, driven by a range of factors. Crucially, effective staffing fell dramatically when health worker absence from facilities was incorporated. Health worker absence from facilities was highest at the smallest facilities and at public facilities. We observed large variation in caseloads, including several countries with per worker daily caseloads of under 16 (i.e. one or fewer cases per half hour in an eight-hour shift). Facilities in rural and urban areas had similar caseloads per effective clinical health worker. This suggests that in many settings, at current patient levels, there is no shortage in the quantity of health workers. Furthermore, the large variation within countries suggests that much of the shortage may be due to inefficiencies or challenges in deployment. While staffing availability may not be a major constraint in many settings, can patients get the care they need? We found that a significant proportion of medical workers scored zero on a series of vignettes about important maternal and child health conditions, from 0.4% of doctors and clinical officers in Malawi to 24.3% in Niger, with higher numbers for nurses and midwives in each country as compared with doctors.

Health staffing challenges in African health systems are complex, multi-faceted and unique to individual country contexts, as demonstrated by the significant between- and within-country variation across all human resources for health performance measures. However, some challenges are ubiquitous: health workers lack basic clinical competencies, caseloads are imbalanced, and there is substantial absence of workers from health facilities; all of these contribute to sub-standard service delivery to patients and inefficient health systems. With expanding populations and large unmet needs, health systems in Africa will require a significant expansion of the health workforce. However, widespread staffing shortages do not appear to be the principal constraint in the short term.

The study complements previous analyses of the SDI surveys. Di Giorgio *et al*. (2020) focused on overall health centre readiness, finding that—on average across countries—the probability that a patient visiting a health centre would find at least one staff present with both the competency and essential inputs required to provide child, neonatal and maternity care is just 14%. Daniels *et al.* (forthcoming) find that most of the variation in competency is within countries and within professional cadres. Andrews *et al*. (2021) demonstrated the variation in the quality of care both between and within communities, which is substantial. Gatti *et al*. (2021) provided an overview analysis of health worker absence from facilities, caseloads, health worker ability and infrastructure (including equipment, supplies and medicine). Daniels *et al.* (2024) examined caseloads across facilities, identifying a high concentration: the average facility has fewer than ten patients per day, but ten percent of facilities account for half of the total patients. This study adds a comprehensive focus on health workers, including a demonstration of where the data show dramatic staffing gaps (e.g. facilities with only one or even zero clinical health workers) and adjusting staffing and caseloads for health worker absence from facilities, which significantly shifts the results relative to previous works.

### Implications of these findings

Three key findings from this study merit highlighting as they have substantial implications for improving health outcomes. First, primary healthcare facilities, the backbone of universal health coverage and a primary access point for the most vulnerable population groups, are the most affected by health workforce challenges. Many frontline facilities lack clinical staff and have the least qualified health workers. This has important implications for the quality of primary care and the way health systems are designed. Efforts to enhance the efficiency of the health sector can only be successful if preceded by the establishment of high-quality primary care services. A strong primary care system is even more important considering the challenges related to the emigration of highly skilled health professionals to countries with perceived better work and life conditions and the negative impacts in terms of availability of such specialists at higher levels of care. At the same time, in some countries, the possibility of emigration can lead to higher domestic investments in human capital (Abarcar and Theoharides, 2024), so the net effect on the domestic stock of health workers is not always negative. The loss of health workers is a challenge affecting health systems in the Africa region, yet it does not change the findings of this study, which instead focuses on the unavailability of providers who have a current assignment in country.

Second, although there is noteworthy variation across countries and health worker cadres, the starkest finding is the low level of competence of health workers. This finding is aligned with The Lancet Global Health Commission on High Quality Health Systems, which found that poor quality care is common across conditions and countries (Kruk *et al*., 2018). This has implications for both the stock and flow of health workers. For existing cadres, creative solutions are needed to improve the quality of care, including new models for in-service training, use of technology to support diagnosis and treatment and supportive supervision. These findings also suggest that there is a crisis in medical and nursing education. Addressing this will also require systemic changes, including reforms of curricula and pedagogical approaches, leveraging technology, improved oversight of both public and private training institutions and stricter competency requirements for graduating students. A related implication of our findings is that given the low caseload, a health workforce strategy cannot be devised in isolation of understanding and addressing the reasons for low levels of utilization of health services, including poor physical access, financial barriers, low quality and lack of trust.

Third, while on average health worker caseload varied substantially both across and within countries, we did not find significant differences across facility types, managing authority, and in between urban and rural facilities. This could be because caseload is driven by different competing reasons resulting in a net null effect. For example, demand for services may be higher in urban areas but that is also where most health workers prefer to work and where the private market is flourishing, giving more options to patients to seek care and more options to providers to increase their income by working in both the public and private sectors. On the contrary, rural, small health facilities have few providers, but demand might also be limited due to low confidence in the quality of care provided—including the unavailability of higher skilled providers, limited service availability or restricted operating hours.

Finally, we did not see a single facility type or health worker cadre have consistently higher health worker absence from facilities, and unauthorized absence was very low (under 10%). These findings indicate that high health worker absence from facilities is a human resource management issue that must be improved if countries are to provide the best possible quality care to patients. While activities beyond providing health services at the health facility are an important part of a health worker’s job, facilities must ensure that quality care can be provided to patients in need.

### Limitations of this work

Some limitations of this study warrant discussion. First, we used health worker caseload as a proxy for workload, acknowledging that this is an imperfect measure of each health worker’s workload, especially in smaller health facilities where health workers are often in charge of administrative tasks. Second, the differences in the way health systems are organized across countries may limit cross-country comparability of this measure. Nevertheless, the large variation in caseload does highlight significant differences that cannot be solely explained by health system differences, for which we control for.

Third, to assess health workers’ clinical competency, we use clinical vignettes, which also present some limitations: they assess what a health worker ‘knows’ about the selected clinical ‘scenarios’ but fail to capture what health workers ‘do in practice’ (Das *et al*., 2008; Leonard and Masatu, 2010). There are trade-offs when it comes to selecting the best methodology to assess health workers’ clinical competency, as some more advanced methods—such as the use of standardized patients—are not suitable for nationally representative studies at scale that allow for cross-country comparison. Hence, the use of clinical vignettes is a useful methodology to enable measurement, monitoring and comparability of health workers’ clinical competencies. Fourth, our data were limited in fully understanding the reasons for health worker absence from facilities. Further research is warranted to gain a better understanding of reasons for health worker absence from facilities including the impact of facility versus community-based service provision strategies on health worker absence. For example, if one facility assigns more workers to visit households in the community, absence from facilities may appear higher in those communities despite high delivery of health services. Fifth, we had limited data from hospitals and the vast majority of the data were from primary care facilities. Finally, our analysis consists of a series of descriptive statistics and correlations. It can serve as a complement to quasi-experimental and experimental work to understand how changing factors in health systems translate into better outcomes for patients. All of these limitations point to the need for richer data and further analysis as health systems seek to improve services.

## Conclusions

This analysis demonstrates how SDI data may be useful to explore health workforce challenges. It also points to future research that can help countries develop evidence-informed health workforce strategies and policies, such as the need to better understand the low demand for health care and the bottlenecks to improve health workers’ clinical capacity, combined with the role played by human resource policies and regulations, and real-world decision-making on staffing presence/absence from facilities and how these impact the demand for care and the quality of care received by patients, among others. It highlights the need for nimble approaches to routinely monitor health worker performance through fast and cost-effective data collection and analysis methods to provide timely information to decision makers. Such timely data are also paramount to better identify effective interventions to improve the availability and quality of health workers in the region and monitor progress over time against pre-determined health worker performance objectives. Future efforts are needed to further study staffing challenges in SSA including investigating (1) the know-do gap; (2) the cyclical causality of low staffing, high health worker absence from facilities, low competency and low demand/caseload levels; and (3) the best interventions to reduce health worker absence from facilities and increase competency overall, both by improving the production and retainment of health workers.

As countries continue to strive for universal health coverage, solutions to health staffing challenges will become increasingly vital for success. Our findings highlight the need to shift focus from simply counting the number of health workers to routinely monitoring the number of ‘skilled health workers working efficiently to deliver quality health services to the population’ to make substantial gains towards solving the health workforce crisis in Africa. There is an opportunity for countries to reconfigure health systems for greater efficiency and quality, but this will require a greater evidence base for country planning, since each country faces unique health workforce challenges.

## Supplementary Material

czae034_Supp

## Data Availability

The datasets generated and/or analysed during the current study are available in the World Bank data repository, https://microdata.worldbank.org/index.php/catalog/sdi

## Footnotes

1. According to the World Development Indicators, SSA had 0.2 physicians and just 1 nurse or midwife per 1000 people (2017–18, the most recent date for which data are available). For South Asia—the region with the next lowest concentration of medical workers—those numbers are 0.8 and 1.5, respectively.

2. For the comparison of our 10 study countries with SSA as a whole, see Di Giorgio *et al*. (2020) and Supplementary Table S2.

3. For the purposes of this analysis, the term ‘health worker’ refers to all cadres of staff in a facility (e.g. medical staff, nursing staff and community health workers based at the facility). It does not include non-medical staff or community health workers based elsewhere but that report to a health facility. In addition, the term ‘clinical staff’ refers to cadres of health workers who are expected to provide clinical care, more specifically doctors, clinical officers, nurses and midwives. It does not include cadres of health workers not expected to provide clinical care such as pharmacists and laboratory technicians. For each country, the specific cadres of health workers included as clinical staff differ.

4. Not all clinical staff were assessed for health worker absence from the facility (up to a total of 10 health workers were assessed for absence per facility). We explored two alternative methods of adjusting for health worker absence from the facility. In the first, we took the total number of clinical staff and reduced that by the number of clinical staff absent. Since not all clinical staff were assessed, this method would undercount absence. In the second, we did the same as in the first, but we restricted the analysis to the subset of facilities where all clinical staff were assessed for absence from the facility (i.e. mostly smaller facilities). Results for the sensitivity analyses are presented in Supplementary Table S10. The preferred method (in the main text) allows for a reduction in proportion to the actual absence of clinical staff.

5. Malawi has community level village clinics which report data on service utilization to the health centre level. The health centre aggregates that data into their monthly reporting forms. This may have led to inflation in the number of outpatient services delivered at the primary level. While there is evidence of low health worker density and high service utilization as an ongoing problem in Malawi, we could not confirm the validity of the caseload data and therefore have excluded it from the analysis.

6. Hospital includes hospitals of all levels (district, regional and national).

7. Health centre includes facilities designed to run as outpatient clinics and may include inpatient treatment or a maternity ward. These are facilities characterized by higher-level staff with the ability to diagnose and treat a range of health conditions and responsibility for a larger catchment area (e.g. health centre, level 2 basic health centre and integrated health centre).

8. Health post includes facilities designed to run as outpatient clinics treating common diseases and offering antenatal care (e.g. dispensary, level 1 basic health centre and health hut).

9. The combined nurse/midwife category was created considering the different staff categorization between countries and to allow for cross-country comparability. The ‘other health workers’ category was created for health workers not expected to provide clinical care such as laboratory technicians, pharmacists, public health officers and health records information officers.

10. Survey weights were calculated for facilities as the inverse of the probability of selection for the facility inventory. Survey weights were calculated for health workers separately for health worker absence and health worker competency. Health worker absence weights were calculated as the inverse of the probability of selection for the health worker absence assessment based on the total number of medical staff recorded on the staff roster times the facility weight. Competency weights were calculated as the inverse of the probability of selection for the competency vignette based on the total number of medical staff who see outpatients recorded on the staff roster times the facility weight. Facility-level sample weights were unavailable for Mozambique, so unweighted facility-level results are reported.

11. For the caseload regression, mean competency scores were missing for 11 facilities (0.2% of data). These missing values were imputed using the country average mean competency score.

12. We have chosen not to disaggregate results for facilities with 0 versus 1 clinical health worker as both situations highlight a staffing challenge of concern.

13. Zero clinical staff does not mean zero staff. Some facilities have no staff who are classified as those authorized to treat patients, but in practice, some other staff may treat patients.

14. As with any non-experimental regression analysis, gender differences are correlations, not causal indicators, and may be correlated both with variables that are controlled for in the regression and with variables that are not controlled for.

## Reflexivity statement

The authors include six women and five men who span multiple levels of seniority. The authors are based in academic institutions and two international organizations. The authors are a multi-cultural, multi-national team, including an author who is Senegalese and based in Côte d’Ivoire, and all authors have extensive experience working, including conducting SDI surveys, in SSA. Our authors do not include an individual from each low- and middle-income country represented in this analysis as the analysis for this study was conducted through the World Bank headquarters to explore cross-country findings from the SDI surveys.
