# Supplementary material for: Human resource challenges in health systems: evidence from 10 African countries
Source: Health Policy Plan. 2024 May 6;39(7):693–709. doi: 10.1093/heapol/czae034 (PMC11308604; doi:10.1093/heapol/czae034)
Supplement: czae034_Supp [file czae034_supp.zip › SDI_manuscript_supplementary tables_FINAL_06Nov2023_clean.pdf]

Supplementary Information

Supplementary Table S1: Key characteristics of selected countries and comparisons by income level and region

Country	World Bank income level[a]	Population, total[a]	GDP (current US$)[a]	Current health expenditure (% of GDP) [a]	Maternal mortality ratio (modeled estimate, per 100,000 live births)[a]	Infant mortality rate (per 1,000 live births)[a]	Human Development Index (HDI)[1,b]	HDI Rank[2,b]
Kenya	Lower middle income	51,985,780	100,657,505,601	4.29	530	29.00	0.575	152
Madagascar	Low income	28,225,177	13,051,441,203	3.88	392	45.30	0.501	173
Malawi	Low income	19,377,061	12,056,108,780	5.43	381	32.10	0.512	169
Mozambique	Low income	31,178,239	14,156,864,916	7.62	127	52.50	0.446	185
Niger	Low income	24,333,639	13,744,174,582	6.20	441	59.90	0.400	189
Nigeria	Lower middle income	208,327,405	432,198,936,002	3.38	1047	72.30	0.535	163
Sierra Leone	Low income	8,233,970	4,062,906,364	8.76	443	80.50	0.477	181
Tanzania	Lower middle income	61,704,518	66,068,737,757	3.75	238	34.90	0.549	160
Togo	Low income	8,442,580	7,389,329,393	5.96	399	44.50	0.539	162
Uganda	Low income	44,404,611	37,605,430,214	3.96	284	32.10	0.525	166
Low income countries		667,053,670	436,035,519,309	5.14	409	48.50		
Lower middle income countries		3,117,225,754	6,619,062,047,764	3.91	255	34.00		
Upper middle income countries		2,766,467,575	23,928,189,369,749	6.10	61	9.80		
High income countries		1,241,726,323	53,937,976,780,898	14.02	12	4.20		
Sub-Saharan Africa		1,151,302,081	1,714,053,357,043	4.92	536	51.10	0.547	
World		7,820,963,775	85,215,150,558,552	10.89	223	28.90	0.732	

[1]Human Development Index is a composite index measuring average achievement in three basic dimensions of human development—a long and healthy life, knowledge, and a decent standard of living. The HDI can be interpreted as follows: Very high human development = 0.896, High human development = 0.754, Medium human development = 0.636, Low human development = 0.518.
[2]HDI rank is based on countries with data; ranges from 1-191

Data sources:
a The World Bank, World Development Indicators (2020). https://databank.worldbank.org/source/world-development-indicators#
b UNDP, The Human Development Index (2021). https://hdr.undp.org/data-center/human-development-index#/indicies/HDI

Supplementary Table S2: Total number of facilities, health workers, health workers assessed for absence from facilities, and health workers assessed for competency, by facility type, managing authority, urban/rural, cadre, and country

	Kenya	Madagascar	Malawi	Mozambique	Niger	Nigeria	Sierra Leone	Tanzania	Togo	Uganda	Total
**Facilities**											
Facility type											
Hospital	285	37	101	38	16	411	30	30	16	9	**973**
Health center	594	316	847	157	67	1458	109	91	46	133	**3818**
Health post	2159	91	158	0	172	516	397	262	118	252	**4125**
Managing authority											
Private/NGO	1276	155	531	2	35	182	43	117	37	158	**2536**
Public	1762	289	575	193	220	2203	493	266	143	236	**6380**
Urban/rural											
Urban	789	226	306	23	63	950	159	161	54	109	**2840**
Rural	2249	218	800	172	192	1435	377	222	126	285	**6076**
**Total**	**3038**	**444**	**1106**	**195**	**255**	**2385**	**536**	**383**	**180**	**394**	**8916**
**Health workers**											
Facility type											
Hospital	10381	517	3770	1733	606	9463	1706	1244	360	114	**29894**
Health center	5971	1522	8793	1239	416	10654	1190	2216	564	1359	**33924**
Health post	8052	161	727	0	309	1201	2159	1700	440	874	**15623**
Managing authority											
Private/NGO	8692	968	4722	57	153	1535	590	2035	326	842	**19920**
Public	15712	1232	8568	2915	1178	19783	4465	3125	1038	1505	**59521**
Urban/rural											
Urban	10247	1697	3916	623	1010	12362	3050	3341	746	791	**37783**
Rural	14157	503	9374	2349	321	8956	2005	1819	618	1556	**41658**
Cadre											
Doctor/ Clinical officer	4408	683	1228	1023	94	960	62	1181	142	381	**10162**
Nurse/ Midwife	11134	1084	2039	1242	5066	1553	1874	609	1067	29424	**5066**
Other	8861	433	9659	707	15169	3440	2090	613	893	39346	**15169**
**Total**	**24404**	**2200**	**13290**	**2972**	**1331**	**21318**	**5055**	**5160**	**1364**	**2347**	**79441**
**Health workers - Absenteeism**											
Facility type											
Hospital	1832	245	802	322	126	2417	227	227	137	30	**6365**
Health center	3549	1160	3138	614	270	6237	604	676	352	625	**17225**
Health post	6885	160	365	0	265	1110	1256	1216	428	621	**12306**
Managing authority											
Private/NGO	4790	617	1653	16	101	668	258	731	238	413	**9485**
Public	7476	948	2652	920	560	9096	1829	1388	679	863	**26411**
Urban/rural											
Urban	3644	1084	1131	168	345	4569	946	1087	382	283	**13639**
Rural	8622	481	3174	768	316	5195	1141	1032	535	993	**22257**
Cadre											
Doctor/ Clinical officer	2111	525	424	423	53	371	23	481	82	290	**4783**
Nurse/ Midwife	5638	729	1771	305	351	1624	553	745	388	621	**12725**
Other	4517	311	2110	208	257	7765	1511	890	447	361	**18377**
**Total**	**12266**	**1565**	**4305**	**936**	**661**	**9764**	**2087**	**2119**	**917**	**1276**	**35896**
**Health workers- Competency**											
Facility type											
Hospital	586	70	425	278	121	1097	84	70	46	16	**2793**
Health center	1032	454	1018	416	178	3231	217	134	110	314	**7104**
Health post	2867	95	79	0	215	686	528	294	147	399	**5310**
Managing authority											
Private/NGO	1697	211	680	11	58	320	94	152	67	242	**3532**
Public	2788	408	842	683	456	4694	735	346	236	487	**11675**
Urban/rural											
Urban	1178	343	520	130	248	2337	343	241	121	162	**5623**
Rural	3307	276	1002	564	266	2677	486	257	182	567	**9584**
Cadre											
Doctor/ Clinical officer	1764	350	441	283	35	462	30	366	64	186	**3981**
Nurse/ Midwife	2624	256	1001	288	263	919	264	86	211	367	**6279**
Other	97	13	80	123	216	3631	535	46	28	173	**4942**
**Total**	**4485**	**619**	**1522**	**694**	**514**	**5014**	**829**	**498**	**303**	**729**	**15207**

Notes: All country surveys were representative at the national level except for Nigeria, which, owing to security concerns, covered 12 of 36 states and was representative only at the state level. In addition, several surveys were sub-nationally representative. In Kenya, data were collected to be representative at the county level and in Malawi, a census of facilities was selected for the survey. The surveys used a multistage cluster-sampling approach to ensure that data were representative of rural and urban areas, private and public facilities, and facility type (i.e., primary vs. secondary).

The difference in the number of health workers assessed for absence from health facilities compared to competency is related to eligibility criteria for inclusion in each assessment. All health workers were eligible for the assessment of health worker absences whereas only health workers offering outpatient consultations were eligible for the health worker competency assessment. In many smaller facilities, the scenario arose where there were at least ten health workers on the roster to meet the sampling needs for assessment of health worker absences, but only a fraction of those health workers provided outpatient consultations. As a result, the total number of health workers included in the assessment of health worker absences is more than twice as large as the number of health workers included in the competency assessment.

Supplementary Table S3: Diagnostic accuracy and treatment accuracy detailed definitions

Disease	Diagnostic accuracy	Treatment accuracy
Diarrhea with dehydration	The integrated management of childhood illness (IMCI) guidelines suggest that this case should be classified as diarrhea with severe dehydration due to the presence of three warning signs: lethargy, sunken eyes, and skin pinch going back very slowly.[1] Clinicians should arrive at the dual diagnosis of diarrhea and dehydration but are allowed a broader range of classifications than suggested by the IMCI guidelines. These diagnoses include any mention of diarrhea ("diarrhea" or "acute diarrhea") and any mention of dehydration ("dehydration," "moderate dehydration," or "severe dehydration"). In Mozambique and Niger, the child was listed as unable to drink or drinking poorly, another risk sign for severe dehydration. The way that the diagnosis for diarrhea with dehydration was recorded varied slightly from country-to country. For example, Nigeria and Uganda listed "acute diarrhea with severe dehydration" as the only possible diagnosis. Madagascar listed "diarrhea with moderate dehydration" and "diarrhea with severe dehydration." Togo listed "diarrhea with severe dehydration." All of these answers are counted as correct. For these countries, it is not possible to provide an accurate estimate of how many doctors diagnosed diarrhea alone; it is only possible to calculate the joint diagnosis rate.	The World Health Organization (WHO) guidelines on the integrated management of childhood illness (IMCI) note that the correct treatment of diarrhea with severe dehydration is to give intravenous fluid immediately, to insert a nasogastric (NG) tube if that is not possible, and to refer the patient to a higher-level facility if neither treatment is available. If the child has only some dehydration, then oral rehydration salts (ORS) are the recommended treatment. Given the symptoms, the correct treatment should be rehydration with an intravenous (IV) line or an NG tube. However, the use of ORS plus zinc is also counted as correct. Because the child was able to drink in most vignettes, the providers may have incorrectly believed that the dehydration was less severe. Correct treatment rates would be much lower if only IV fluids or an NG tube was counted as correct (13% correct). Uganda did not include an option for ORS, so only treatment with IV fluids or an NG tube is taken as a correct response. Kenya did not include an option for IV fluids or NG tube, so only ORS with zinc is taken as a correct response.
Pneumonia	A diagnosis of pneumonia is counted as the correct response.	The IMCI guidelines suggest oral amoxicillin for five days as treatment for pneumonia. Severe pneumonia can be treated with "the first dose of an appropriate antibiotic" and urgent referral to a hospital. In addition, children had a fever of 38.5 in the vignette, and IMCI guidelines recommend an

[1] World Health Organization. 2014. "IMCI Chart Booklet." Geneva, Switzerland. https://www.who.int/docs/default-source/mca-documents/imci-chart-booklet. pdf?sfvrsn=f63af425_1&download=true.

Disease	Diagnostic accuracy	Treatment accuracy
		antipyretic in this case. Correct treatment is counted as treatment with amoxicillin, a first-line antibiotic, and any antipyretic.
Diabetes	Based on the characteristics presented in this vignette, a diagnosis of type 2 diabetes is the correct response. However, the option of diabetes (type not specified) was available in Kenya, Madagascar, Mozambique, Niger, Sierra Leone, and Tanzania. Clinicians were not prompted to select a specific type if they answered diabetes. Although treatment varies for different types of diabetes, the general diabetes response is also classified as correct. This practice results in higher correct diagnosis rates, an improvement by 14–50 percentage points depending on the country.	The WHO package of essential noncommunicable disease interventions (PEN) protocols states, "Individuals with persistent fasting blood glucose >6 mmol/l despite diet control should be given metformin and/or insulin as appropriate."[2] Correct treatment is counted as any hypoglycemic (including insulin) or referral to a specialist. Referral to a higher level is the recommended protocol for diabetes at the primary level in multiple countries so that option is counted as correct. Although PEN protocol suggests diet control before prescribing hypoglycemics, here prescriptions on first presentation are counted as correct.
Tuberculosis	A diagnosis of tuberculosis is counted as the correct response.	The WHO guidelines for treatment of tuberculosis recommend combination therapy, ideally with a fixed-dose combination.[3] Providers are simply required to mention combination therapy. Knowledge of correct duration and dosage is not necessary, and providers would score worse if this knowledge were required. For example, 23% of providers prescribed combination therapy, but only 8% accurately recalled the correct dosage and timing (this comparison is possible in Madagascar, Mozambique, Niger, Sierra Leone, and Tanzania). However, Nigeria and Uganda recorded "correct duration and dose" as one option, so providers are assessed on having gotten the correct dosage and timing. This likely creates a downward bias for provider treatment abilities in these two countries. Kenya did not record

[2] World Health Organization. 2019. "WHO Package of Essential Noncommunicable (PEN) Disease Interventions: Noncommunicable Diseases and Their Risk Factors." Geneva, Switzerland. www.who.int/ncds/management/pen_tools/en/.
[3] World Health Organization. 2018. "Guidelines for Treatment of Drug-Susceptible Tuberculosis and Patient Care (2017 Update)." Geneva, Switzerland. www.who.int/tb/publications/2017 /dstb_guidance_2017/en/

Disease	Diagnostic accuracy	Treatment accuracy
		any information on whether providers got the correct duration and dosage.
Malaria with anemia	This case should be classified as malaria with anemia, and clinicians are required to arrive at this dual diagnosis. All countries except Togo included "malaria" as an option, and all countries except Nigeria and Uganda included "simple malaria" as an option. Both of these diagnoses are counted as correct. All countries also included "severe malaria" as an option, and this nonspecific diagnosis is not counted as correct because the case does not meet the definition of severe, and severe malaria would require different treatment. Anemia was listed simply as "anemia" without specifying severity. Providers therefore received credit for specifying malaria or simple malaria and anemia. Kenya excluded this module entirely, so it is omitted and its diagnostic accuracy is counted as the average of the four other vignettes.	IMCI guidelines recommend that children with a positive malaria test should be given "recommended first-line antimalarial" and "one dose of paracetamol in clinic" for fever reduction. In addition, iron should be given for treatment of anemia. The questions on malaria treatment varied a bit between countries, and credit is given for treatment with any artemisinin combination therapy or artemether-lumefantrin (coartem). In addition to antimalarials, the provider must prescribe paracetamol and iron for the anemia. Kenya did not include the malaria vignette and is excluded. Nigeria and Uganda did not include questions about iron and are excluded for the sake of comparability.

Supplementary Table S4: Additional facility level measures detailed definitions

Domain	Item	Definition
Infrastructure	Improved sanitation	Credit is given if facility reports and enumerator confirms facility has one or more functioning flush toilets or ventilated improved pit (VIP) latrines, or covered pit latrine (with slab).
Infrastructure	Improved water source	Credit is given if a facility reports their main source of water is piped into the facility, piped onto facility grounds, or comes from a public tap/standpipe, tubewell/borehole, a protected dug well, a protected spring, bottled water, or a tanker truck. This definition is based on the WHO/UNICEF Joint Monitoring Program for Water Supply, Sanitation and Hygiene.
Infrastructure	Electricity	Credit is given if facility reports using electric power grid, fuel-operated generator, battery-operated generator, or a solar powered system as their main source of electricity.
Equipment	Thermometer	Credit is given if a facility reports and the enumerator observes that the facility has one or more functioning thermometers (used for measuring patient body temperature).
Equipment	Stethoscope	Credit is given if a facility reports and the enumerator observes that the facility has one or more functioning stethoscopes.
Equipment	Sphygmomanometer	Credit is given if a facility reports and the enumerator observes that the facility has one or more functioning sphygmomanometers.
Equipment	Weighing scale	Credit is given if a facility reports and the enumerator observes that the facility has one or more functioning adult, child or infant weighing scale.

Supplementary Table S5: Staffing norms by cadre of clinical staff and health facility type

		Doctor	Clinical officer	Doctor/clinical officer	Nurse/Midwife	Nurse	Midwife	**Total**
Kenya	Health center	2	11		37			**50**
	Dispensary	0	2		8			**10**
Malawi	Urban health center			12	14			**26**
	Rural health center			3	8			**11**
Nigeria	Health center	0	0		3			**3**
Niger	Health center II	1			6			**7**
	Health center I	0			4			**4**
	Health post	0			1			**1**
Uganda	Health center IV	2	4		11			**17**
	Health center III	0	2		6			**8**
	Health center II	0	0		2			**2**
Tanzania	Health center (minimum number)	2	2		10			**14**
	Health center (maximum number)	2	3		15			**20**
	Dispensary (minimum number)	0	1		4			**5**
	Dispensary (maximum number)	0	2		7			**9**
Togo	Primary healthcare center I	0				2	1	**3**
	Primary healthcare center II	1				4	3	**8**

Notes: Roman numerals after facility types refer to the size of the facility (i.e., health center II is larger than health center I). Staffing norms were not available for all countries.

Sources: These numbers are drawn from the following documents (see the reference list at the end of this supplementary appendix for more information): Kenya Ministry of Health (2014); Malawi Ministry of Health and Population (2018); Nigeria Federal Ministry of Health (2007); Ministère de la Santé Publique du Niger (2016); Uganda Ministry of Public Service (No date); Tanzania Ministry of Health and Social Welfare (2013); Ministère de la Santé du Togo (2013).

Supplementary Table S6: Average staffing norms compared to actual staffing levels

	EXPECTED average number of clinical staff	ACTUAL average number of clinical staff in HCs and HPs
Kenya	30	3.35
Malawi	18.5	3.37
Nigeria	3	0.58
Niger	5.5	1.38
Uganda	12.5	3.42
Togo	5.5	2.6
Tanzania	12	4.36

Notes: The "expected" average number of clinical staff is the average across cadres and facility types from Supplementary Table S5. The "actual" number of clinical staff is from Table 3.
Source: Authors' construction.

Supplementary Table S7: Health worker absence from facilities - total, by country (% and 95% CI)

	Kenya	Madagascar	Malawi	Mozambique	Niger	Nigeria	Sierra Leone	Tanzania	Togo	Uganda	All
National	53.8 (52.0 - 55.6)	26.0 (22.5 - 29.6)	36.5 (34.0 - 39.1)	23.3 (19.0 - 27.6)	31.1 (26.4 - 35.9)	34.4 (32.5 - 36.3)	39.9 (36.8 - 42.9)	13.8 (11.0 - 16.5)	43.8 (37.1 - 50.5)	43.8 (38.7 - 48.9)	34.7 (26.3 - 43)
Facility type											
Hospital	58.9 (55.0 - 62.8)	19.2 (13.0 - 25.5)	43.3 (37.6 – 49.0)	23.9 (18.0 - 29.8)	38.2 (27.1 - 49.4)	30.3 (26.6 - 34.1)	34.0 (26.4 - 41.5)	12.6 (6.6 - 18.6)	47.6 (38.9 - 56.4)	27.7 (6.0 - 49.4)	33.6 (23.6 - 43.5)
Health center	52.3 (50.0 - 54.6)	29.1 (24.3 - 33.9)	34.3 (32.2 - 36.5)	22.3 (16.7 - 27.9)	35.0 (28.1 – 42.0)	38.2 (36.5 - 39.9)	39.0 (33.2 - 44.8)	14.9 (9.4 - 20.3)	40.9 (31.1 - 50.8)	46.2 (38.9 - 53.5)	35.2 (27.4 - 43.1)
Health post	48.5 (47.0 - 49.9)	24.0 (14.3 - 33.6)	9.2 (5.4 - 12.9)	NA	20.4 (13.5 - 27.3)	32.5 (29.3 - 35.6)	43.0 (39.1 - 46.9)	13.7 (11.2 - 16.3)	45.9 (35.7 - 56.1)	43.5 (37.6 - 49.4)	31.2 (19.8 - 42.6)
Managing authority											
Private/NGO	46.3 (43.6 – 49.0)	25.5 (20.4 - 30.5)	30.7 (27.8 - 33.7)	11.4 (0.0 - 30.2)	34.7 (22.2 - 47.2)	19.6 (15.4 - 23.7)	33.9 (26.6 - 41.3)	11.9 (8.0 - 15.8)	44.5 (33.2 - 55.8)	38.7 (31.0 - 46.5)	29.7 (20.8 - 38.6)
Public	58.6 (56.2 - 60.9)	26.3 (21.6 - 31.1)	39.2 (35.8 - 42.7)	23.6 (19.2 - 27.9)	30.9 (25.9 - 35.9)	38.9 (37.0 - 40.8)	40.8 (37.5 - 44.1)	14.9 (11.2 - 18.6)	43.0 (35.8 - 50.3)	47.5 (40.9 - 54.2)	36.4 (27.3 - 45.5)
Urban/Rural											
Urban	55.1 (51.8 - 58.4)	24.5 (21.0 - 28.1)	43.1 (37.5 - 48.7)	23.2 (13.2 - 33.2)	34.8 (28.0 - 41.5)	33.4 (30.5 - 36.3)	37.8 (33.5 – 42.0)	14.3 (10.3 - 18.2)	45.5 (35.9 - 55.1)	36.5 (26.9 - 46.1)	34.8 (26.3 - 43.3)
Rural	52.6 (50.8 - 54.4)	27.7 (21.3 - 34.2)	32.5 (30.4 - 34.6)	23.4 (19.0 - 27.9)	27.6 (21.1 - 34.2)	35.8 (33.8 - 37.8)	42.7 (38.5 - 46.9)	12.8 (10.1 - 15.4)	41.0 (32.7 - 49.3)	49.3 (45.0 - 53.7)	34.6 (25.8 - 43.3)
Health worker cadre											
Doctor/ Clinical officer	56.1 (51.8 - 60.5)	32.6 (25.5 - 39.6)	41.4 (31.8 – 51.0)	24.3 (18.2 - 30.4)	38.3 (20.4 - 56.2)	28.9 (21.4 - 36.5)	24.9 (3.9 – 46.0)	21.1 (13.6 - 28.6)	58.2 (37.0 - 79.4)	42.5 (31.7 - 53.3)	36.8 (27.6 - 46.1)
Nurse / Midwife	55.8 (52.9 - 58.8)	24.9 (19.9 - 29.9)	33.1 (29.2 – 37.0)	24.7 (17.3 - 32.1)	32.3 (25.9 - 38.6)	38.7 (33.1 - 44.4)	31.9 (26.2 - 37.6)	10.5 (7.4 - 13.5)	48.4 (38.3 - 58.5)	47.7 (41.9 - 53.5)	34.8 (25.2 - 44.4)
Other worker	49.6 (47.2 - 52.1)	18.5 (12.8 - 24.1)	38.8 (36.2 - 41.3)	18.8 (11.5 - 26.2)	28.2 (20.5 - 35.9)	33.6 (31.9 - 35.3)	43.7 (40.0 - 47.3)	12.3 (7.9 - 16.6)	36.5 (26.9 – 46.0)	38.3 (32.1 - 44.5)	31.8 (23.2 - 40.5)

Supplementary Table S8: Health worker absence from facilities - unauthorized, by country (% and 95% CI)

	Kenya	Madagascar	Malawi	Mozambique	Niger	Nigeria	Sierra Leone	Tanzania	Togo	Uganda	All
National	2.9 (2.4 - 3.5)	1.8 (0.6 – 3.0)	2.1 (1.6 - 2.5)	1.1 (0.4 - 1.8)	5.1 (2.5 - 7.8)	3.8 (3.3 - 4.3)	4.8 (3.4 - 6.2)	0.1 (0.0 - 0.3)	7.9 (4.8 - 10.9)	8.9 (6.5 - 11.3)	3.9 (1.8 - 5.9)
Facility type											
Hospital	2.3 (1.2 - 3.4)	0.8 (0.0 - 1.8)	0.3 (0.0 - 0.6)	0.7 (0.0 - 1.5)	4.1 (0.0 - 8.2)	2.6 (1.8 - 3.4)	3.2 (0.4 – 6.0)	0.2 (0.0 - 0.5)	12.0 (6.3 - 17.8)	4.0 (0.0 – 9.0)	3.0 (0.5 - 5.5)
Health center	2.6 (2.0 - 3.2)	1.9 (0.5 - 3.2)	3.2 (2.5 - 3.9)	1.9 (0.5 - 3.2)	3.3 (0.0 - 6.7)	4.7 (4.0 - 5.4)	5.5 (2.6 - 8.4)	0.1 (0.0 - 0.4)	9.0 (3.0 - 14.9)	10.3 (6.6 - 14.1)	4.3 (1.9 - 6.6)
Health post	3.9 (3.4 - 4.5)	3.4 (0.0 - 9.5)	2.2 (0.0 - 4.8)	NA	8.8 (2.9 - 14.7)	5.1 (3.6 - 6.5)	5.0 (3.0 – 7.0)	0.1 (0.0 - 0.3)	6.3 (3.6 – 9.0)	7.6 (4.7 - 10.4)	4.7 (2.6 - 6.8)
Managing authority											
Private/NGO	1.6 (1.3 - 1.9)	0.5 (0.0 - 1)	1.3 (0.8 - 1.9)	0.0 (0.0 – 0.0)	1.9 (0.0 - 4.3)	2.2 (0.8 - 3.5)	1.9 (0.0 – 4.0)	0.0 (0.0 - 0.1)	7.7 (2.2 - 13.3)	3.9 (1.5 - 6.3)	2.1 (0.5 - 3.7)
Public	3.8 (2.9 - 4.6)	2.5 (0.7 - 4.3)	2.4 (1.8 – 3.0)	1.1 (0.4 - 1.9)	5.4 (2.6 - 8.1)	4.3 (3.8 - 4.8)	5.2 (3.6 - 6.9)	0.2 (0.0. - 0.4)	8.0 (5.5 - 10.5)	12.5 (8.8 - 16.3)	4.5 (2.0 - 7.1)
Urban/Rural											
Urban	2.4 (1.4 - 3.4)	1.2 (0.6 - 1.8)	0.8 (0.3 - 1.3)	1.3 (0.0 - 2.8)	1.8 (0.2 - 3.3)	3.2 (2.5 – 4.0)	4.8 (2.9 - 6.8)	0.1 (0.0 - 0.3)	7.5 (2.9 – 12.0)	5.6 (1.2 - 9.9)	2.9 (1.2 - 4.6)
Rural	3.5 (3.0 - 3.9)	2.5 (0.1 - 4.9)	2.8 (2.2 - 3.4)	1.0 (0.2 - 1.8)	8.4 (3.6 - 13.2)	4.6 (3.9 - 5.2)	4.7 (2.5 - 6.9)	0.2 (0.0 - 0.5)	8.5 (5.4 - 11.7)	11.4 (8.8 - 13.9)	4.8 (2.2 - 7.3)
Health worker cadre											
Doctor/ Clinical officer	2.8 (1.5 - 4.2)	1.1 (0.2 – 2.0)	0.1 (0.0 - 0.3)	1.2 (0.2 - 2.2)	4.7 (0.0 - 12.2)	0.5 (0.0 – 1.0)	0.0 (0.0 – 0.0)	0.0 (0.0 – 0.0)	3.4 (0.0 - 7.8)	8.8 (3.7 - 13.8)	2.3 (0.3 - 4.3)
Nurse / Midwife	2.0 (1.6 - 2.4)	2.7 (0.4 – 5.0)	1.0 (0.6 - 1.5)	0.7 (0.0 - 1.4)	3.9 (0.5 - 7.2)	2.0 (1.2 - 2.8)	3.9 (2.0 - 5.8)	0.2 (0.0 - 0.5)	9.9 (4.4 - 15.4)	9.8 (6.8 - 12.8)	3.6 (1.1 - 6.1)
Other worker	4.3 (3.1 - 5.5)	0.8 (0.0 - 1.5)	4.1 (3.1 – 5.0)	1.2 (0.0 - 2.6)	7.0 (2.3 - 11.7)	4.8 (4.1 - 5.4)	5.3 (3.4 - 7.2)	0.2 (0.0 - 0.4)	6.8 (3.0 - 10.7)	7.1 (4.5 - 9.8)	4.2 (2.3 – 6.0)

Supplementary Table S9: Reasons for health worker absence from facilities, by country (% and 95% CI)

	Kenya	Madagascar	Malawi	Mozambique	Niger	Nigeria	Sierra Leone	Tanzania	Togo	Uganda	All
Authorized absence	40.5 (37.8 - 43.2)	35.7 (28.4 - 43.1)	22.9 (18.7 - 27.1)	26.3 (17.0 - 35.7)	34.0 (25.7 - 42.4)	32.7 (29.8 - 35.5)	17.5 (13.8 - 21.2)	29.6 (21.1 – 38.0)	26.8 (18.3 - 35.3)	24.2 (18.6 - 29.8)	30.0 (22.5 - 37.4)
Official mission	8.7 (7.6 - 9.8)	13.1 (7.9 - 18.3)	7.9 (5.1 - 10.7)	20.8 (12.7 – 29.0)	6.1 (2.5 - 9.7)	15.9 (13.8 - 17.9)	8.2 (5.7 - 10.8)	4.0 (2.0 - 5.9)	11.0 (3.6 - 18.5)	11.4 (6.6 - 16.2)	11.5 (6.6 - 16.4)
Outreach or fieldwork	1.4 (1.1 - 1.7)	0.2 (0.0. - 0.7)	21.9 (19.0 - 24.8)	0.0 (0.0 – 0.0)	0.0 (0.0 – 0.0)	2.8 (2.1 - 3.5)	11.2 (9.0 - 13.4)	50.0 (0.0 - 12.5)	0.0 (0.0 - 0.0)	3.5 (2.0 - 5.0)	5.4 (2.3 - 13.1)
Sick or on maternity leave	5.2 (4.1 - 6.3)	5.1 (2.9 - 7.2)	3.9 (2.4 - 5.3)	14.7 (7.6 - 21.9)	19.7 (12.2 - 27.1)	11.1 (9.3 - 12.9)	9.1 (6.9 - 11.4)	23.5 (15.3 - 31.6)	9.4 (2.7 – 16.0)	7.9 (5.2 - 10.6)	9.8 (4.4 - 15.2)
Training, seminar, meeting	7.7 (6.2 - 9.2)	24.8 (18.7 - 30.8)	18.5 (14.5 - 22.5)	21.4 (11.7 - 31.1)	11.3 (6.0 - 16.6)	8.8 (7.3 - 10.3)	6.3 (3.7 – 9.0)	35.7 (24.5 - 46.9)	34.7 (23.6 - 45.8)	30.6 (25.2 – 36.0)	14.1 (7.3 - 20.9)
Unauthorized absence	5.4 (4.5 - 6.4)	7.1 (2.6 - 11.5)	5.6 (4.4 - 6.9)	4.7 (1.7 - 7.8)	16.5 (8.8 - 24.3)	11.4 (9.9 - 12.9)	12.0 (8.6 - 15.4)	1.1 (0.0 - 2.1)	18.0 (11.1 - 24.9)	21.9 (16.7 - 27.2)	9.0 (4.9 - 13.1)
Other	31.0 (28.7 - 33.3)	14.0 (10.0 - 18.1)	19.3 (15.2 - 23.5)	12.0 (6.2 - 17.8)	12.3 (6.5 - 18.2)	17.4 (13.2 - 21.7)	35.5 (31.5 - 39.6)	1.2 (0.3 - 2.2)	0.2 (0.0 - 0.4)	0.4 (0.1 - 0.8)	20.2 (11.6 - 28.9)

Supplementary Table S10: Clinical staffing patterns in health centers and health posts adjusted for health worker absence from facilities, by country (number/proportion and 95% CI)

		Kenya	Madagascar	Malawi	Mozambique	Niger	Nigeria	Sierra Leone	Tanzania	Togo	Uganda	All
		n = 2753	n = 407	n = 1005	n = 157	n = 239	n = 1974	n = 506	n = 353	n = 164	n = 385	n = 7943
Proportion of health centers and health posts with one or fewer clinical staff adjusted for health worker absence from facilities	A: Total number of clinical staff reduced by proportion of clinical staff absent	63.9 (61.9 - 65.8)	69.2 (61.4 – 77.0)	39.4 (36.4 - 42.4)	47.8 (39.9 - 55.6)	85.3 (80.4 - 90.3)	92.4 (91.0 - 93.7)	84.4 (80.7 - 88.2)	31.0 (24.3 - 37.8)	68.9 (54.3 - 83.5)	59.0 (52.8 - 65.2)	64.1 (49.6 - 78.7)
	B: Total number of clinical staff reduced by subtracting number of clinical staff absent	63.1 (61.1 – 65.0)	69.2 (61.5 – 77.0)	36.2 (33.2 - 39.2)	47.8 (39.9 - 55.6)	85.3 (80.4 - 90.3)	91.8 (90.4 - 93.2)	84.2 (80.4 - 87.9)	30.9 (24.1 - 37.7)	68.3 (53.7 – 83.0)	56.2 (49.9 - 62.5)	63.3 (48.5 - 78.1)
	B1: Subset of facilities where all clinical staff were assessed for absence from facility	n = 2238	n = 341	n = 117	n = 331	n = 97	n = 308	n = 206	n = 135	n = 183	n = 214	n = 4170
		75.8 (73.9 - 77.8)	73.2 (65.2 - 81.2)	61.3 (56.1 - 66.6)	56.4 (47.4 - 65.4)	79.7 (70.3 - 89.2)	89.8 (85.7 - 93.9)	81.6 (75.6 - 87.6)	41.8 (32.3 - 51.3)	70.7 (54.4 – 87.0)	80.0 (73.8 - 86.3)	71.1 (60.9 - 81.2)
Proportion of health centers and health posts with two or fewer clinical staff adjusted for health worker absence from facilities	A: Total number of clinical staff reduced by proportion of clinical staff absent	82.7 (81.2 - 84.3)	89.3 (85.1 - 93.5)	61.6 (58.6 - 64.6)	67.5 (60.2 - 74.9)	91.9 (88.4 - 95.4)	95.6 (94.5 - 96.7)	91.7 (88.7 - 94.8)	60.6 (53.0 - 68.2)	79.7 (67.3 - 92.1)	72.7 (66.8 - 78.6)	79.3 (70.0 - 88.7)
	B: Total number of clinical staff reduced by subtracting number of clinical staff absent	81.0 (79.4 - 82.6)	88.9 (84.7 - 93.2)	58.1 (55.1 - 61.2)	67.5 (60.2 - 74.9)	91.2 (87.4 - 94.9)	94.7 (93.5 - 95.9)	91.2 (88.1 - 94.2)	59.8 (52.2 - 67.4)	79.7 (67.3 - 92.1)	68.1 (62.0 - 74.1)	78.0 (68.2 - 87.8)
	B1: Subset of facilities where all clinical staff were assessed for absence from facility	n = 2238	n = 341	n = 117	n = 331	n = 97	n = 308	n = 206	n = 135	n = 183	n = 214	n = 4170
		93.7 (92.6 - 94.8)	93.8 (89.9 - 97.6)	88.5 (85.1 – 92.0)	82.1 (75.1 – 89.0)	92.9 (87.3 - 98.5)	96.8 (94.0 - 99.6)	95.7 (92.7 - 98.7)	82.9 (76.5 - 89.3)	83.0 (69.4 - 96.6)	93.4 (89.8 - 97.1)	90.3 (86.2 - 94.3)

Note: This table presents clinical staffing adjusted for health worker absence from facilities using two alternative methods of adjustment, as outlined in endnote 5 of the main paper.

Supplementary Table S11: Average health worker caseload per facility, by country (number and 95% CI)

	Kenya	Madagascar	Malawi	Mozambique	Niger	Nigeria	Sierra Leone	Tanzania	Togo	Uganda	All
National	23.1 (21.9 - 24.2)	6.4 (5.0 - 7.7)	47.5 (43.7 - 51.3)	23.2 (20.0 - 26.5)	10.9 (8.6 - 13.2)	2.8 (2.5 - 3.2)	11.3 (10.2 - 12.5)	11.7 (9.6 - 13.8)	6.3 (4.8 - 7.9)	17.0 (14.2 - 19.7)	16.0 (6.7 - 25.3)
Facility type											
Hospital	24.6 (19.9 - 29.2)	5.5 (3.1 - 7.8)	27.2 (19.9 - 34.5)	16.7 (7.9 - 25.5)	1.1 (0.6 - 1.7)	4.1 (2.9 - 5.4)	8.0 (6.3 - 9.8)	33.5 (5.5 - 61.4)	6.6 (4.0 - 9.3)	16.6 (2.9 - 30.2)	14.4 (6.5 - 22.3)
Health center	25.0 (22.4 - 27.5)	7.7 (5.5 - 9.9)	49.5 (45.2 - 53.9)	24.9 (21.5 - 28.3)	21.6 (17.1 - 26.2)	2.7 (2.3 – 3.0)	11.3 (8.7 - 13.9)	14.2 (7.6 - 20.9)	7.6 (4.5 - 10.6)	17.1 (11.8 - 22.4)	18.2 (8.6 - 27.7)
Health post	22.2 (21.0 - 23.5)	4.0 (2.8 - 5.2)	64.2 (49.1 - 79.3)	NA	6.1 (4.5 - 7.6)	2.0 (1.7 - 2.3)	11.5 (10.1 - 12.9)	10.4 (8.5 - 12.4)	5.8 (4.1 - 7.6)	16.9 (13.7 - 20.2)	15.9 (1.1 - 30.7)
Managing authority											
Private/NGO	15.0 (13.5 - 16.6)	11.8 (9.8 - 13.7)	23.8 (19.5 - 28.1)	12.5 (0.0 - 29)	50 (2.6 - 7.4)	3.2 (1.9 - 4.6)	9.2 (7.1 - 11.3)	11.3 (7.0 - 15.6)	6.8 (4.3 - 9.4)	7.0 (4.7 - 9.4)	10.6 (6.3 - 14.8)
Public	30.4 (28.9 - 32)	5.1 (3.6 - 6.6)	58.0 (53.2 - 62.8)	23.4 (20.0 - 26.7)	11.2 (8.7 - 13.6)	2.7 (2.5 – 3.0)	11.5 (10.2 - 12.8)	11.9 (9.4 - 14.3)	6.0. (4.0 – 8.0)	26.1 (22.0 - 30.3)	18.6 (6.7 - 30.6)
Urban/Rural											
Urban	20.1 (17.5 - 22.6)	10.5 (8.7 - 12.3)	36.7 (27.2 - 46.1)	19.6 (5.6 - 33.6)	8.1 (4.5 - 11.7)	3.2 (2.5 - 3.9)	10.5 (8.5 - 12.5)	15.3 (9.7 - 20.9)	6.6 (4.3 – 9.0)	10.1 (5.7 - 14.6)	14.1 (7.2 - 20.9)
Rural	24.6 (23.4 - 25.7)	4.9 (3.3 - 6.6)	49.3 (45.2 - 53.5)	23.7 (20.5 - 26.9)	11.2 (8.7 - 13.8)	2.6 (2.3 - 2.9)	11.7 (10.2 - 13.2)	10.1 (8.4 - 11.8)	6.1 (4.0 - 8.2)	20.2 (16.8 - 23.5)	16.4 (6.5 - 26.4)

Supplementary Table S12: Proportion of facilities with low and high caseload, by country (% and 95% CI)

	Kenya	Madagascar	Malawi	Mozambique	Niger	Nigeria	Sierra Leone	Tanzania	Togo	Uganda	All
**Proportion of facilities with a caseload of <5**											
Private/NGO	31.7 (28.7 - 34.6)	30.7 (19.7 - 41.8)	22.1 (15.3 - 28.8)	50.0 (0 - 119.9)	70.5 (52.6 - 88.3)	83.1 (76.2 - 89.9)	32.5 (16.8 - 48.1)	40.5 (24.7 - 56.4)	53.2 (23.3 - 83.2)	64.1 (54.1 - 74.2)	47.8 (33.6 - 62.1)
Public	5.0 (3.8 - 6.1)	73.1 (64.8 - 81.5)	2.4 (0.8 - 4.1)	13.6 (8.6 - 18.6)	40.7 (30.3 - 51.1)	86.5 (84.7 - 88.3)	32.3 (27.6 - 37.1)	35.6 (26.7 - 44.5)	63.9 (50.2 - 77.7)	3.4 (0.5 - 6.3)	35.7 (13.8 - 57.5)
Total	17.8 (16.1 - 19.4)	65.2 (57 - 73.4)	8.5 (5.9 - 11)	14.0 (9 - 19)	41.8 (31.8 - 51.8)	85.9 (84 - 87.8)	32.4 (27.8 - 36.9)	37.0 (29.2 - 44.7)	59.5 (44.9 - 74.1)	32.5 (25.6 - 39.4)	39.4 (21.9 - 57)
**Proportion of facilities with a caseload of >=30**											
Private/NGO	12.8 (10.5 - 15.1)	9.1 (4.3 - 13.9)	26.2 (19.0 - 33.4)	0.0 (0.0 – 0.0)	0.0 (0.0 – 0.0)	1.9 (0.0 - 4.4)	0.0 (0.0 – 0.0)	3.7 (0.1 - 7.3)	0.0 (0.0 – 0.0)	4.7 (0.7 - 8.7)	5.8 (0.0 - 11.8)
Public	36.1 (33.4 - 38.7)	1.2 (0.0 - 2.3)	68.6 (63.6 - 73.6)	27.2 (20.7 - 33.7)	10.7 (4.1 - 17.2)	0.8 (0.3 - 1.3)	6.2 (4.1 - 8.4)	6.5 (3.8 - 9.2)	3.8 (0.0 - 11.2)	29.8 (22.4 - 37.2)	19.1 (3.6 - 34.5)
Total	24.9 (23.1 - 26.8)	2.6 (1.3 – 4.0)	55.6 (51.1 - 60.1)	26.9 (20.5 - 33.3)	10.3 (4.0 - 16.5)	1.0 (0.4 - 1.6)	5.7 (3.7 - 7.7)	5.7 (3.5 - 7.9)	2.2 (0.0 - 6.6)	17.8 (13.2 - 22.3)	15.3 (3.1 - 27.4)

Supplementary Table S13: Linear regression of caseload on facility characteristics

	**Model 1** – facility characteristics on caseload		**Model 2** – Model 1 + readiness		**Model 3** – Model 1 + readiness + absenteeism		**Model 4** – Model 1 + readiness + absenteeism + competency	
**Country (REF = Kenya)**								
Madagascar	-15.4	***	-14.4	***	-10.3	***	-9.0	***
Malawi	21.9	***	22.6	***	26.5	***	26.2	***
Mozambique	-6.3	***	-5.6	**	-0.2		1.9	
Niger	-17.5	***	-16.4	***	-12.3	***	-9.9	***
Nigeria	-25.9	***	-24.4	***	-21.4	***	-18.7	***
Sierra Leone	-14.8	***	-14.2	***	-12.6	***	-11.3	***
Tanzania	-10.7	***	-10.6	***	-5.9	***	-5.3	***
Togo	-19.9	***	-19.6	***	-18.0	***	-16.6	***
Uganda	-4.8	***	-4.4	***	-4.2	***	-2.3	*
**Facility type (REF = Hospital)**								
Health center	0.1		0.5		0.7		1.2	
Health post	-1.6		-0.7		1.5		2.3	*
**Managing authority (REF = Private/ NGO)**								
Public	12.8	***	13.2	***	11.7	***	11.5	***
**Urban/Rural (REF = Urban)**								
Rural	-1.3	*	-0.9		-0.5		-0.5	
**Equipment availability**			0.01	**	0.01		0.01	
**Infrastructure availability**			0.02	***	0.02	**	0.01	*
**Health worker absence from facilities**					20.1	***	20.2	***
**Provider competency**							0.1	***
**(Intercept)**	17.8	***	14.0	***	6.1	***	2.3	
**R-squared**	0.2884		0.2901		0.3359		0.3397	

Significance codes: '***' 0.001, '**' 0.01, '*' 0.05

Note: N = 7236; 1680 facilities excluded due to missing data on caseload. Reasons for missing caseload data include:

1) The facility was missing data on the number of staff
2) The facility was missing data on the number of outpatient visits
3) The caseload value was set to missing/omitted because it was greater than 200 which was deemed to be unrealistically high

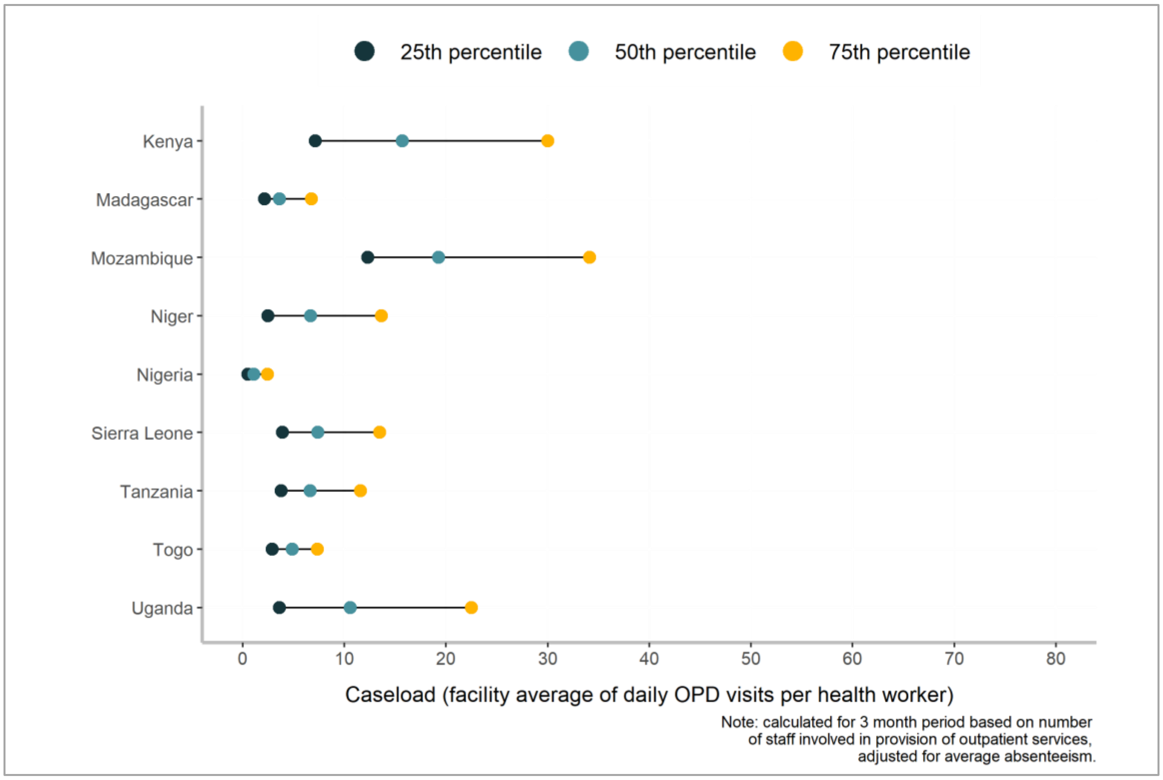

Supplementary Figure S1a: Caseload distribution for health centers and health posts, by country

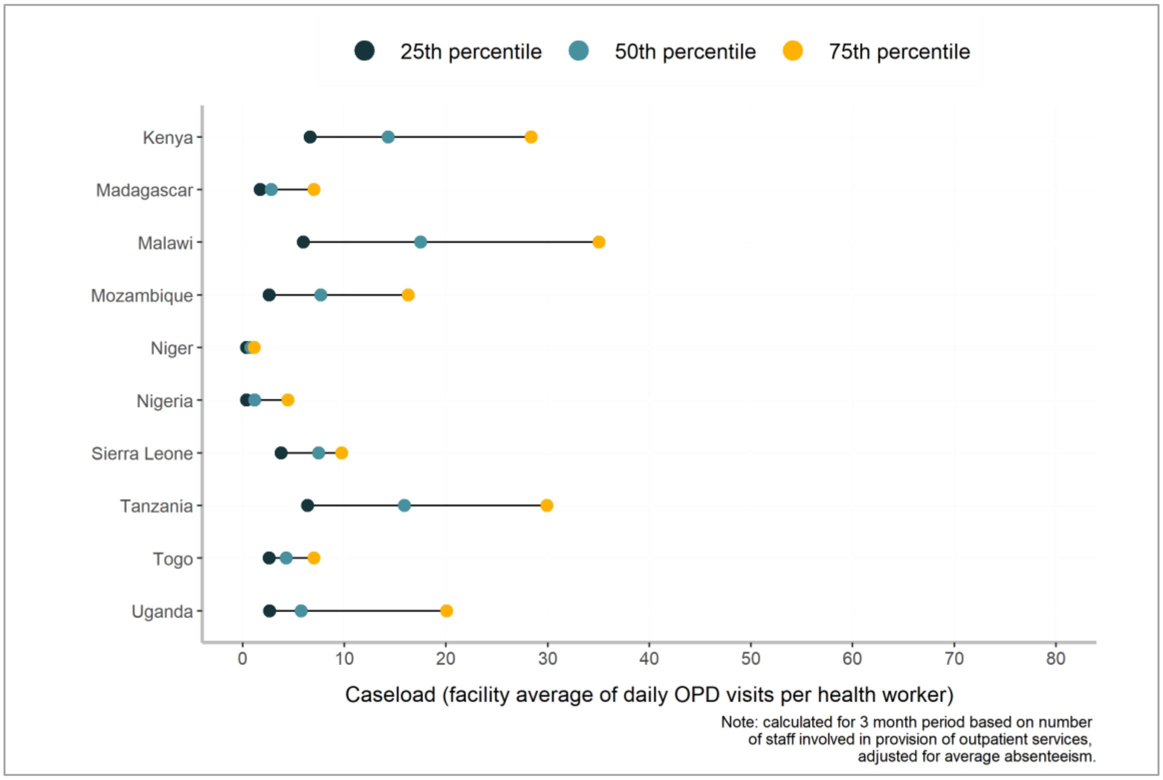

Supplementary Figure S1b: Caseload distribution for hospitals, by country

Supplementary Table S14: Health worker diagnostic accuracy, by country (% and 95% CI)

	Kenya	Madagascar	Malawi	Mozambique	Niger	Nigeria	Sierra Leone	Tanzania	Togo	Uganda	All
National	66.8 (65.6 – 68.0)	48.1 (43.3 - 52.9)	71.5 (69.6 - 73.5)	58.4 (56.0 - 60.7)	39.9 (37.4 - 42.4)	40.9 (38.6 - 43.3)	49.1 (47.0 - 51.2)	69.2 (64.4 – 74.0)	50.3 (45.1 - 55.6)	56.3 (52.7 – 60.0)	55.1 (47.0 - 63.2)
Facility type											
Hospital	70.6 (66.8 - 74.4)	54.5 (50.3 - 58.7)	74.3 (71.9 - 76.8)	62.4 (59.5 - 65.4)	44.7 (39.6 - 49.9)	52.0 (47.4 - 56.7)	60.5 (54.7 - 66.3)	71.5 (63.3 - 79.6)	50.1 (41.5 - 58.7)	71.3 (60.2 - 82.3)	61.2 (53.7 - 68.7)
Health center	67.1 (65.6 - 68.6)	49.9 (43.5 - 56.4)	67.5 (64.6 - 70.5)	49.6 (46.2 - 53.0)	44.3 (40.9 - 47.6)	31.7 (30.4 – 33.0)	54.1 (50.3 – 58.0)	75.1 (65.3 - 84.8)	56.3 (47.9 - 64.7)	64.2 (60.1 - 68.3)	56.0 (46.7 - 65.2)
Health post	64.5 (63.5 - 65.4)	38.5 (31.4 - 45.5)	54.7 (47.8 - 61.7)	NA	30.3 (26.4 - 34.3)	29.3 (27.1 - 31.5)	45.4 (42.9 - 47.9)	56.8 (51.7 – 62.0)	45.3 (38.9 - 51.8)	40.8 (37.2 - 44.5)	45.1 (35.9 - 54.2)
Managing authority											
Private/NGO	65.4 (64.1 - 66.6)	51.7 (45.8 - 57.6)	67.6 (65.4 - 69.9)	59.7 (44.7 - 74.8)	46.2 (40.9 - 51.6)	52.6 (46.2 – 59.0)	56.8 (51.7 – 62.0)	74.6 (64.4 - 84.8)	54.5 (44.2 - 64.8)	56.2 (51.3 - 61.1)	58.5 (52.5 - 64.6)
Public	67.6 (65.9 - 69.3)	46.9 (40.9 - 52.9)	73.0 (70.6 - 75.5)	58.3 (55.9 - 60.7)	39.5 (36.9 - 42.1)	38.4 (36.3 - 40.4)	48.1 (45.9 - 50.3)	66.8 (61.4 - 72.1)	46.9 (41.2 - 52.5)	56.4 (51.3 - 61.5)	54.2 (45.5 - 62.9)
Urban/Rural											
Urban	67.1 (64.5 - 69.7)	52.1 (47.7 - 56.5)	74.6 (71.9 - 77.4)	67.4 (63.4 - 71.3)	46.5 (43.4 - 49.7)	44.4 (40.8 – 48.0)	54.3 (51.2 - 57.4)	74.2 (68.5 - 79.9)	53.2 (45.5 - 60.9)	64.6 (59.4 - 69.9)	59.8 (51.9 - 67.8)
Rural	66.6 (65.7 - 67.5)	45.9 (39.0 - 52.8)	67.4 (65.0 - 69.8)	54.4 (51.9 - 56.9)	34.1 (30.7 - 37.4)	36.5 (33.7 - 39.2)	45.1 (42.4 - 47.7)	56.0 (49.6 - 62.5)	45.7 (38.2 - 53.3)	49.9 (46.6 - 53.2)	50.2 (42.1 - 58.2)
Health worker cadre											
Doctor/ Clinical officer	70.6 (68.5 - 72.7)	57.4 (54.1 - 60.6)	74.6 (71.6 - 77.6)	63.3 (60.2 - 66.3)	57.2 (51.2 - 63.1)	57.6 (50.4 - 64.8)	59.3 (49.4 - 69.1)	72.6 (67.0 - 78.2)	64.3 (54.5 - 74.1)	71.7 (67.4 - 75.9)	64.8 (59.9 - 69.8)
Nurse / Midwife	63.0 (62.0 - 64.1)	41.1 (33.5 - 48.6)	70.0 (67.4 - 72.5)	51.7 (48.3 - 55.1)	43.2 (39.9 - 46.4)	44.4 (41.1 - 47.8)	52.0 (48.6 - 55.4)	45.7 (39.4 - 52.1)	46.3 (40.8 - 51.8)	50.2 (46.8 - 53.5)	50.8 (44.2 - 57.3)
Other worker	48.5 (41.1 - 55.9)	30.2 (16.4 – 44.0)	54.4 (42.2 - 66.6)	53.7 (46.8 - 60.6)	32.7 (29.0 - 36.4)	30.2 (28.8 - 31.7)	47.5 (44.9 - 50.1)	46.5 (37.7 - 55.2)	39.2 (24.1 - 54.4)	28.7 (24.3 – 33.0)	41.2 (33.9 - 48.4)

Supplementary Table S15: Health worker treatment accuracy, by country (% and 95% CI)

	Kenya	Madagascar	Malawi	Mozambique	Niger	Nigeria	Sierra Leone	Tanzania	Togo	Uganda	All
National	74.4 (73.1 - 75.6)	55.9 (51.4 - 60.3)	77.1 (75.3 - 78.8)	46.3 (43.2 - 49.4)	41.0 (38.6 - 43.5)	35.0 (32.9 - 37.1)	57.4 (55.6 - 59.2)	70.1 (66.9 - 73.3)	55.9 (49.5 - 62.4)	47.9 (43.7 - 52.2)	56.1 (46.0 - 66.2)
Facility type											
Hospital	78.9 (75.1 - 82.6)	61.6 (50.2 – 73.0)	79.2 (76.7 - 81.7)	49.5 (45.3 - 53.7)	43.8 (37.7 - 49.8)	47.7 (43.9 - 51.5)	66.4 (60.3 - 72.5)	75.8 (71.1 - 80.5)	56.5 (48.4 - 64.6)	71.4 (67.2 - 75.6)	63.1 (53.6 - 72.6)
Health center	74.4 (72.4 - 76.4)	56.8 (51.1 - 62.5)	74.0 (71.8 - 76.2)	39.5 (36.2 - 42.8)	41.1 (37.3 - 44.9)	24.4 (23.0 - 25.8)	59.6 (56.1 - 63.1)	74.6 (69.4 - 79.7)	65.1 (56.7 - 73.5)	56.9 (52.4 - 61.5)	56.7 (44.5 - 68.8)
Health post	71.8 (70.7 - 72.8)	49.6 (43.1 - 56.1)	64.2 (56.4 - 71.9)	NA	39.6 (35.9 - 43.3)	21.4 (19.3 - 23.5)	55.2 (53.0 - 57.4)	52.1 (48.3 – 56.0)	48.0 (39.5 - 56.5)	28.2 (24.9 - 31.6)	47.8 (35.4 - 60.2)
Managing authority											
Private/NGO	68.7 (67.0 - 70.3)	49.4 (40.5 - 58.2)	75.3 (72.6 – 78.0)	50.8 (40.4 - 61.2)	44.4 (36.9 - 51.9)	46.0 (39.7 - 52.3)	62.3 (57.2 - 67.4)	69.8 (65.0 - 74.6)	57.8 (45.6 – 70.0)	47.3 (39.4 - 55.2)	57.2 (49.1 - 65.2)
Public	77.6 (75.9 - 79.3)	58.0 (52.9 - 63.1)	77.7 (75.5 – 80.0)	46.2 (43.1 - 49.4)	40.8 (38.2 - 43.4)	32.6 (30.7 - 34.4)	56.7 (54.8 - 58.6)	70.3 (66.2 - 74.3)	54.3 (47.6 – 61.0)	48.3 (43.4 - 53.3)	56.3 (45.4 - 67.1)
Urban/Rural											
Urban	73.6 (70.7 - 76.5)	51.0 (45.0 – 57.0)	78.8 (76.2 - 81.5)	52.7 (45.4 – 60.0)	40.0 (36.1 - 43.9)	38.7 (35.7 - 41.8)	60.6 (57.9 - 63.4)	75.3 (72.1 - 78.6)	58.8 (49.6 – 68.0)	58.3 (52.0 - 64.6)	58.8 (48.8 - 68.8)
Rural	74.8 (73.9 - 75.8)	58.6 (52.5 - 64.6)	74.7 (72.6 - 76.8)	43.5 (40.7 - 46.3)	42.0 (38.8 - 45.2)	30.1 (27.3 - 32.9)	54.8 (52.4 - 57.2)	56.3 (51.7 – 61.0)	51.3 (41.7 - 60.8)	39.9 (36.5 - 43.3)	52.6 (42.2 - 63.0)
Health worker cadre											
Doctor/ Clinical officer	77.2 (75.0 - 79.4)	60.0 (54.2 - 65.8)	79.5 (76.6 - 82.4)	51.9 (47.4 - 56.5)	46.3 (30.8 - 61.8)	54.2 (48.8 - 59.7)	67.5 (56.0 – 79.0)	73.9 (70.7 - 77.1)	66.3 (57.3 - 75.3)	64.6 (59.3 – 70.0)	64.2 (56.2 - 72.1)
Nurse / Midwife	71.9 (70.8 – 73.0)	53.0 (46.6 - 59.4)	76.4 (74.1 - 78.6)	38.6 (34.5 - 42.7)	41.0 (37.6 - 44.5)	40.5 (36.4 - 44.6)	59.6 (56.2 - 62.9)	46.8 (39.9 - 53.8)	54.0 (46.0 – 62.0)	39.5 (34.5 - 44.5)	52.1 (42.4 - 61.9)
Other worker	50.8 (43.8 - 57.7)	40.9 (26.5 - 55.3)	52.9 (43.1 - 62.7)	41.2 (34.7 - 47.7)	40.2 (36.7 - 43.6)	22.1 (20.7 - 23.5)	56.0 (53.9 - 58.2)	39.5 (31.1 - 47.9)	31.2 (22.6 - 39.9)	23.5 (19.2 - 27.8)	39.8 (31.5 - 48.1)

Supplementary Materials References

Kenya Ministry of Health. (2014). *Human Resources For Health Norms and Standards: Guidelines For The Health Sector*. https://www.health.go.ke/wp-content/uploads/2015/09/16th%20october%20WHO%20Norms%20and%20Standarnds%20%20Book.pdf

Malawi Ministry of Health and Population. (2018). *Human Resources for Health Strategic Plan, 2018-2022*.

Ministère de la Santé du Togo. (2013). Normes Sanitaires du Togo.

Ministère de la Santé Publique du Niger. (2016). Normes et Standards des Infrastructures, Equipements et Ressources Humaines du Systeme de Sante.

Nigeria Federal Ministry of Health. (2007). *National Human Resources for Health Strategic Plan, 2008 to 2012*. https://pdf4pro.com/cdn/nigeria-hrhstrategicplan-2008-2012-who-2b01b2.pdf

Tanzania Ministry of Health and Social Welfare. (2013). *Staffing Levels for Ministry of Health and Social Welfare Departments, Health Service Facilities, Health Training Institutions and Agencies, 2014-2019*. https://www.jica.go.jp/project/tanzania/006/materials/ku57pq00001x6jyl-att/REVIEW_STAFFING_LEVEL_2014-01.pdf

Uganda Ministry of Public Service. (No date). Staffing Schedules for Health Centres II, III, and IV.
